# WGCNA reveals key gene modules regulated by the combined treatment of colon cancer with PHY906 and CPT11

**DOI:** 10.1042/BSR20200935

**Published:** 2020-09-02

**Authors:** Shuqin Xing, Yafei Wang, Kaiwen Hu, Fen Wang, Tao Sun, Quanwang Li

**Affiliations:** 1 Department of Oncology, Dongfang Hospital of Beijing University of Chinese Medicine, Beijing 100078, China; 2 Department of Orthopaedics, Dongfang Hospital of Beijing University of Chinese Medicine, Beijing 100078, China

**Keywords:** chemotherapy, Colon cancer, Irinotecan, PHY906, traditional Chinese Medicine

## Abstract

Irinotecan (CPT11) is one of the most effective drugs for treating colon cancer, but its severe side effects limit its application. Recently, a traditional Chinese herbal preparation, named PHY906, has been proved to be effective for improving therapeutic effect and reducing side effects of CPT11. The aim of the present study was to provide novel insight to understand the molecular mechanism underlying PHY906-CPT11 intervention of colon cancer. Based on the GSE25192 dataset, for different three treatments (PHY906, CPT11, and PHY906-CPT11), we screened out differentially expressed genes (DEGs) and constructed a co-expression network by weighted gene co-expression network analysis (WGCNA) to identify hub genes. The key genes of the three treatments were obtained by merging the DEGs and hub genes. For the PHY906-CPT11 treatment, a total of 18 key genes including Eif4e, Prr15, Anxa2, Ddx5, Tardbp, Skint5, Prss12 and Hnrnpa3, were identified. The results of functional enrichment analysis indicated that the key genes associated with PHY906-CPT11 treatment were mainly enriched in ‘superoxide anion generation’ and ‘complement and coagulation cascades’. Finally, we validated the key genes by Gene Expression Profiling Interactive Analysis (GEPIA) and RT-PCR analysis, the results indicated that EIF4E, PRR15, ANXA2, HNRNPA3, NCF1, C3AR1, PFDN2, RGS10, GNG11, and TMSB4X might play an important role in the treatment of colon cancer with PHY906-CPT11. In conclusion, a total of 18 key genes were identified in the present study. These genes showed strong correlation with PHY906-CPT11 treatment in colon cancer, which may help elucidate the underlying molecular mechanism of PHY906-CPT11 treatment in colon cancer.

## Introduction

Colon cancer is a common malignant tumor and the second leading cause of mortality worldwide in 2018 [1]. With the improvement in people’s living standards and changes in diet and other aspects, the incidence of colon cancer has increased year by year. Colon cancer development is related to many factors, such as age, sex, race/ethnicity, genetic makeup, dietary factors, increased BMI, red meat intake, low physical activity, long-term cigarette smoking (30–40 years), low vegetables and fruit consumption [2]. Studies showed that intestinal flora plays an important role in the occurrence and development of colon cancer [3]. Intestinal flora imbalance will cause damage to the intestinal barrier and impaired innate immune function, leading to inflammation and infection. There is mounting evidence that the intestinal flora can be used as biomarkers for cancer diagnosis and prognostication [4–6], which indicated that the intestinal flora will become an important component of future cancer prevention and therapy. In recent years, several studies have shown that colon cancer is closely related to inflammatory bowel disease (IBD) [7], and the relative risk of colon cancers in IBD patients is approximately two- to three-times greater than that in the general population. Additionally, a number of studies have reported the importance of single inflammatory genes in colon cancer [8,9]. For instance, a previous study conducted by Jiang and colleagues identified 14 inflammatory genes with high prognostic significance, and found a novel role of the inflammation–associated network in colon cancer [10]. Based on these, anti-inflammatory drugs have become a new focus of researchers seeking effective drugs for colon cancer.

The therapy of colon cancer includes surgical resection, radiotherapy, chemotherapy, and others. Chemotherapy is an important treatment for colon cancer in all stages. Irinotecan (CPT11), a DNA topoisomerase I inhibitor, does not hinder the binding of topoisomerase I, but converts this ribozyme into a substance that is harmful to DNA. CPT11 is considered as one of the most effective drugs for treating colon cancer [11]. However, the clinical application of CPT11 is often accompanied by a variety of side effects, including neutropenia, bone marrow suppression, and delayed diarrhea [12–14].

Traditional Chinese medicine (TCM) is one of the unique cultural treasures of China, which has gained growing recognition around the world in recent years. PHY906, one of the TCM, is composed of four main herbs, *Scutelleria baicalensis Georgi, Paeonia lactiflora Pall., Glycyrrhiza uralensis Fisch.*, and *Ziziphus jujuba Mill*. PHY906 has been used for over 1800 years for treating gastrointestinal symptoms. Zou and colleagues [15] found that PHY906 exerts a protective effect in IBD by down-regulated production of pro-inflammatory cytokines and suppression of the NF-κB signaling pathway. Several clinical studies have demonstrated that TCM, as an alternative treatment for cancer, can reduce the incidence of bone marrow suppression and gastrointestinal reactions in chemotherapy [16–18]. In addition to mitigate the side effects of chemotherapy, the combination of chemotherapy and TCM also improves the chemosensitivity of diseases. Several Phase I/II clinical trials with encouraging results have been conducted in the United States to explore the toxicity and clinical efficacy of PHY906. PHY906 was shown to provide cytoprotective effects without dampening the anti-tumor activity of CPT11 in colon cancer patients under chemotherapy. A study demonstrated that PHY906 significantly amplifies the effects of CPT-11 in the tumor tissue; combination of PHY906 and CPT11 (PHY906-CPT11) enhanced anti-tumor activity by creating a unique tissue-specific response [19]. However, few studies focused on the mechanism of anti-tumor modulation of the PHY906-CPT11 in chemotherapy.

Currently, microarray technology has been widely applied to elucidate the tumor progression in different conditions. Weighted gene co-expression network analysis (WGCNA) is a new tool that is used to identify co-expressed modules and hub genes. It has been reported that WGCNA can be useful to identify candidate biomarkers or therapeutic targets of various diseases, such as Alzheimer’s Disease [20], psychiatric disorders [21], glioblastoma [22], and colon cancer [23]. At present, it has been widely used to compare differentially expressed genes (DEGs) and help investigating the interactions among genes in different modules [24]. Besides, it is also used to identify rules for predicting survival of patients by investigating the relationship between clinical traits and tissue microarray data [25].

In the present study, we aimed to investigate molecular mechanism of different treatments in colon cancer. We analyzed the microarray datasets of colon cancer treated with PHY906, CPT11, PHY906-CPT11, and Phosphate Buffered Saline (PBS), respectively. DEGs from three different treatments were identified, then WGCNA was constructed to screen hub genes associated with the three treatments. DEGs and hub genes associated with different treatments were intersected and key genes which were significantly associated with the treatment of colon cancer were obtained finally. The functional annotation of genes (DEGs, hub genes, and key genes) were performed to understand the functions of these genes.

## Materials and methods

### Dataset collection

The gene expression profiles of GSE25192 submitted by Wang et al. (2011) [19] were obtained from the GEO database (https://www.ncbi.nlm.nih.gov/geo). The dataset comprised four groups: ten tumor-bearing mice treated with PBS (‘PBS’), nine tumor-bearing mice treated with PHY906 (‘PHY906’), ten tumor-bearing mice treated with CPT11 (‘CPT11’), and nine tumor-bearing mice treated with the combination of PHY-906 and CPT-11 (‘PHY906-CPT11’).

### Screening and functional enrichment analysis of DEGs

The raw file data were preprocessed in ‘limma’ R package [26], including background correction and expression normalization. Under the threshold of FDR < 0.05 and |log2FC| ≥ 1, the ‘limma’ R package was used to screen out the DEGs between PHY906 and PBS, CPT11 and PBS, PHY906-CPT11 and PBS. Then the ‘pheatmap’ R package was used to construct the expression heatmap while the ‘ggpubr’ and ‘ggthemes’ R packages were used to construct the volcano plot of acquired DEGs. Venn diagram was performed by using the online tool jvenn (http://jvenn.toulouse.inra.fr/app/example.html) to overlap the DEGs in PHY906, CPT11, and PHY906-CPT11.

### Construction of WGCNA and key module identification

The data of GSE25192 were used for cluster analysis to detect outliers using standardized connectivity (Z.K) method suggested by WGCNA authors [27], with the threshold Z.K score < −2. Then, the expression data were used as input datasets of WGCNA, and construct the sample dendrogram and trait heatmap after one outlier sample were excluded, and the similar gene expression profiles were divided into different gene modules. To ensure that the network can obey the scale-free criteria without affecting the network connectivity, the present study selected β = 8 as the soft-thresholding.

After determining the soft-thresholding, the network was developed. The adjacency matrix was transformed into a topological overlapping matrix to construct a network, and the gene dendrogram and module colors were established by using the degree of dissimilarity. To further analyze the module, we calculated the dissimilarity of the module eigengenes (MEs), hierarchically clustered the modules, and merged similar modules. In order to evaluate the co-expression similarity among different modules, characteristic adjacency degrees of correlation among each module were calculated. Interaction of different co-expression modules was assessed by flashClust function, and the heatmap was constructed.

After the modules were identified, the ME was summarized by the first principal component of the module expression levels. Module–trait relationships were estimated using the correlation between MEs and clinical treatment, which allowed efficient identification of the relevant modules. To evaluate the correlation strength, we calculated the module significance (MS), which is defined as the average absolute gene significance (GS) of all the genes in the module. The GS was measured as the log10 transformation of the *P*-value (lgP) in the linear regression between gene expression and clinical information. In the WGCNA, modules with the highest MS score among all modules are usually defined as the key module and selected for further analysis.

### Transcription factors prediction and identification of hub genes for the key module

Enrichr (http://amp.pharm.mssm.edu/Enrichr/) is a comprehensive web-based tool that contains 180184 annotated gene sets from 102 gene set libraries. The gene information for the key module was imported into the Enrichr to obtain the interaction between transcription factors (TFs) and their target genes. To reduce the chance of identifying false-positives, we extracted only TFs with consensus target ChEA and ENCODE geneset libraries and with *P*<0.05 as determined by the Fisher exact test. We then used the Cytoscape 3.4.0 software (Cytoscape Consortium, SanDiego, CA, U.S.A.) to visualize the TF-target gene regulatory networks.

Hub genes were screened out using module connectivity, measured by MM ≥ median and clinical trait relationships, measured by GS ≥ median. Then we exported the full weighted network of the key module. Finally, all the obtained genes’ network from the full weighted network of the key module (if there are too many edges, we filtered the edges according to the weight to obtain the suitable number of edges for visualization by cytoscape) were extracted, and a subnetwork of the full weight network of the key module was finally obtained. Furthermore, the subnetworks were then constructed with the plug-in MCODE in the Cytoscape (degree cut-off ≥ 2, node score cut-off ≥ 0.2, K-core ≥ 2, and max depth = 100). Genes identified in the MCODE subnetworks and exhibiting high weight in the co-expression network were chosen as the hub genes for further analysis. The significance of the gene expression in different treatments was examined by the Wilcoxon’s test (*P*<0.05 was considered as significant differences).

### Identification of key genes by merging DEGs with hub genes

The Venn map was constructed by website (http://bioinformatics.psb.ugent.be/webtools/Venn/) for intersection of the obtained data.

### Validation of the key genes

An online tool, Gene Expression Profiling Interactive Analysis (GEPIA, http://gepia.cancer-pku.cn/), was used to analyze differential expression of the key genes and visualize their expression levels in different stages based on TCGA datasets. The overall survival analysis for each key gene was also performed using GEPIA, and the log-rank tests were used to measure the statistically significance (*P*<0.05).

### Cell culture and treatment

Human colon cancer cell line SW480 was seeded in six-well plates (Corning, U.S.A.) at a density of 5 × 10^4^ cells/well. The cells were cultured in Roswell Park Memorial Institute (RPMI)-1640 medium and incubated at 37°C with 5% CO_2_. A total of 10% fetal bovine serum (FBS), 100 U/ml penicillin, and 100 μg/ml streptomycin were contained in the culture media.

The cells were cultured to approximately 85% density and divided into four groups. Four groups of SW480 cells were respectively treated with PBS, PHY906 (500 μg/ml), CPT11 (5 μg/ml), and PHY906-CPT11 (500 μg/ml-5 μg/ml) for 24 h.

### RNA isolation and qRT-PCR

Total RNA was extracted from SW480 that was treated with different treatments respectively by using the TRIzol reagent. The total RNA was used for reverse-transcription into cDNA using a specific kit for reverse transcription (Promega) according to the manufacturer-provided protocol. The qRT-PCR approach was used for amplification and determination of the mRNA expression level with the SYBR Green PCR Master Mix. The mRNA expression levels relative to the controls (GAPDH expression) were obtained using the 2^−ΔΔ*C*_T_^ method [28]. Primer sequences used in the present study were provided in Table 1.

**Table 1:** Primer sequences for the key genes

Gene symbol	Primer forward (5′–3′)	Primer reverse (5′–3′)
*GBP2*	CACAGGCTGCGTAATTGCAG	CTTGCACACAAGGGAACAACA
*EIF4E*	GAAACCACCCCTACTCCTAATCC	AGAGTGCCCATCTGTTCTGTA
*PRR15*	CCGTAGGCGATCAGACTTCC	CCACCCCTCTGAATCAACCC
*ANAX2*	TCTACTGTTCACGAAATCCTGTG	AGTATAGGCTTTGACAGACCCAT
*DDX5*	CAAAGAGCCCTCACGTCTGT	TTTTGTATCCCTCCGCCGAC
*TARDBP*	TGGAGAAGTTCTTATGGTGCAGGTC	TCCATCTATCATATGTCGCTGTGAC
*PRSS12*	ACACTGCCCACAGAAACCAA	AGGGACTAGCATGAGACGGT
*HNRNPA3*	TGAAAGGGGGCAGTTTTGGT	ACTCAGTGAACAGTTCCACATATTC
*PFDN2*	GTGGGCACCTACCTTGGAAA	AGTAAGCCCTCCGTGTTTGG
*NCF1*	GTCCGGTTGTTTGCAACCTC	GCTATGGCCTAGACAGAGCG
*RGS10*	AGCCTCAAGAGCACAGCCAAAT	GCACGTCCTGAGAGGAAATTCCT
*C3AR1*	CCGGTACACGAACACAGGAA	AACAGCGTGAGGACCTTCTG
*GNG11*	TGAAATGAAGCAGGGTCCGA	CACTGAAGGCCACTGGTGAA
*TYROBP*	GGAGGTCTCTGGGAGGTAGA	TAACGCACATAGTCCGGTGG
*TMSB4X*	GGTATTGTGCGTTGCCTTGG	GTGGGTCGGAGCTCACTTTC
*C1QC*	CAGAGGTGGACTCTCACGGT	CAAGGATTGCCTGTGGACCT
*GAPDH*	GGAGCGAGATCCCTCCAAAAT	GGCTGTTGTCATACTTCTCATGG

### GO function and KEGG pathway enrichment analyses of the DEGs, key module, hub genes and key genes

To understand the biological meaning of the DEGs, key module, hub genes and key genes, clusterProfiler (R package) was used for performing GO enrichment and KEGG pathway analyses. *P*-value <0.05 was regarded as significant.

## Results

### Screening and GO function and KEGG pathway enrichment analyses of DEGs

**Figure 1:**
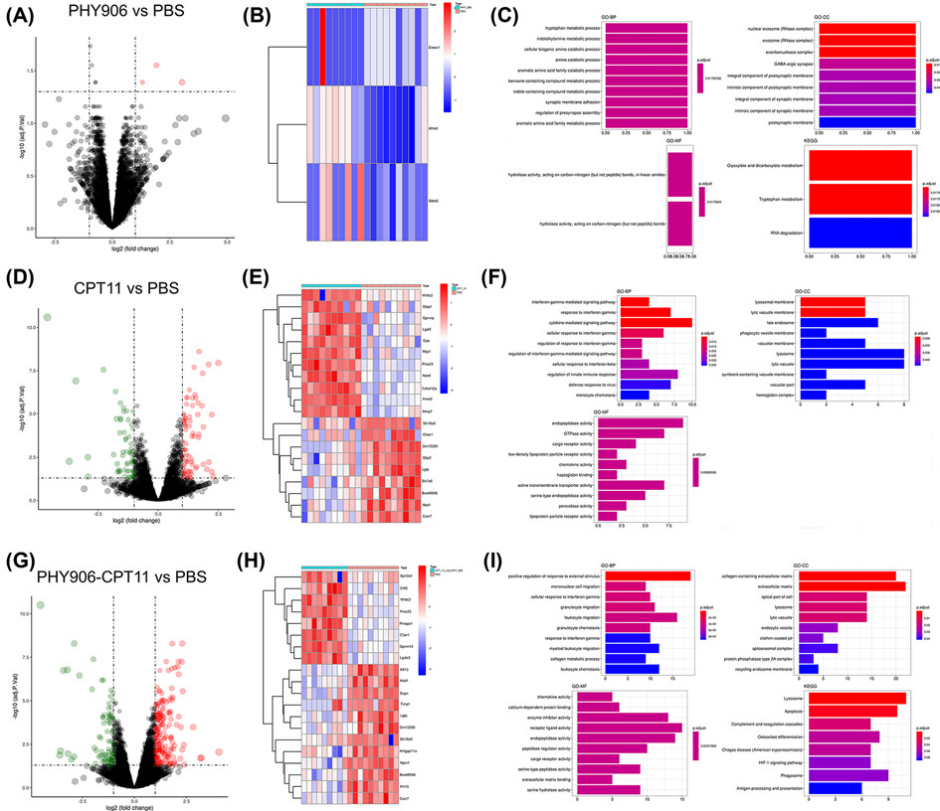
Analysis of DEGs in dataset GSE25192 (**A**) Volcano plot and (**B**) heatmap of DEGs screening between PBS and PHY906. (**C**) GO and pathway analysis of DEGs in group of PBS vs. PHY906. (**D**) Volcano plot and (**E**) heatmap of DEGs screening between PBS and CPT11. (**F**) GO and pathway analysis of DEGs in group of PBS vs. CPT11. (**G**) Volcano plot and (**H**) heatmap of DEGs screening between PBS and PHY906-CPT11. (**I**) GO and pathway analysis of DEGs in group of PBS vs. PHY906-CPT11. Red dots indicate up-regulated genes, and green dots indicate down-regulated genes. X-axis represents the gene number and the represents the GO and KEGG terms. The color represents the −log10 (*P*-value) of each term.

The volcano plots for all genes and the heatmap of the genes with the most significant differential expression were shown in Figure 1. In total, three DEGs were screened out (three up-regulated genes Exosc1, Slitrk2, and Afmid) between PBS and PHY906 (Figure 1A,B), 131 DEGs were screened out (74 up-regulated and 61 down-regulated) between PBS and CPT11 (Figure 1D,F). Genes such as HBB-B1, MYH4, and HBA-A2 were the most down-regulated while Sepp1, Afmid, and Calcb were the most up-regulated in PBS vs. CPT11 comparison. Moreover, 268 DEGs were identified (186 up-regulated and 82 down-regulated) between PBS and PHY906-CPT11, among which Capg, Metrnl, and Calcb were the most up-regulated while Hbb-B1, Hba-A2, and Prss2 were the most down-regulated (Figure 1H,I). Additionally, we found there was only one common gene (Slitrk2) among the DEGs from PHY906, CPT11, and PHY906-CPT11 (Supplementary Figure S1). As shown in Figure 1C, GO analysis revealed that the DEGs between PBS and PHY906 were enriched in different biological processes (BPs) among which tryptophan metabolic process, indolalkylamine metabolic process and cellular biogenic amine catabolic process were the most significantly enriched. In the category of cellular component (GO-CC), these genes were significantly enriched in the functional terms of nuclear exosome, exosome, and exoribonuclease complex. The results also showed that hydrolase activity and acting on carbon–nitrogen (but not peptide) bonds were the most enriched terms in the category of molecular function (GO-MF). For KEGG pathway enrichment analysis, the result showed that these genes were significantly enriched in the functional terms of glyoxylate and dicarboxylate metabolism, and tryptophan metabolism.

The results of the GO function analysis of the DEGs between PBS and CPT11 are listed in Figure 1F. In the category of GO-BP, the genes were significantly enriched in the functional terms of interferon (IFN)-γ-mediated signaling pathway and response to IFN-γ, while in the category of GO-CC, the genes were significantly enriched in the functional terms of lysosomal membrane and lytic vacuole membrane. In the category of GO-MF, these genes were mainly involved in endopeptidase activity, GTPase activity, and so on. There was no enrichment of such genes in KEGG pathway analysis.

The GO function and KEGG pathway enrichment analyses of the DEGs between PBS and PHY906-CPT11 were further performed (Figure 1I). In the category of GO-BP, the genes were significantly enriched in the functional terms of positive regulation of response to external stimulus. The category of GO-CC showed that these DEGs were significantly enriched in collagen-containing extracellular matrix and extracellular matrix. In the category of GO-MF, the DEGs were mainly enriched in chemokine activity, calcium-dependent protein binding, enzyme inhibitor activity, and receptor ligand activity. The results of KEGG analysis showed that these DEGs were mainly enriched in lysosome and apoptosis.

### Construction of WGCNA and key module identification

**Figure 2:**
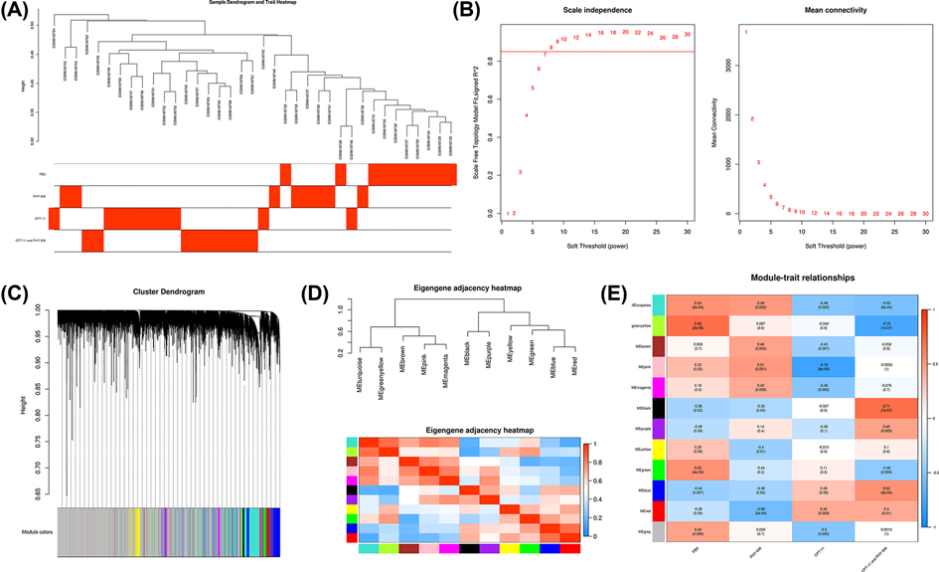
WGCNA (**A**) Sample clustering after excluded GSM618744. (**B**) Determining soft-thresholding power in WGCNA: the scale-free fit index and the mean connectivity for various soft-thresholding powers. (**C**) The cluster dendrogram and color display of co-expression network modules. (**D**) Hierarchical clustering of module hub genes and heatmap plot of the adjacencies in the hub gene network. (**E**) Heatmap of the correlation between module eigengenes and treatments of colon cancer.

After detecting the outliers using Z.K method with the threshold Z.K score < −2, the GSM618744 was excluded (Figure 2A). Next, we performed the analysis of network topology for thresholding powers from 1 to 30 and identified the relatively balanced scale independence and mean connectivity of the WGCNA. As the lowest power for the scale-free topology fit index on 0.85, power value 8 was selected to produce a hierarchical clustering tree (Figure 2B). Then we used the WGCNA to assign genes with similar expression patterns into one module, and 12 gene modules were obtained (Figure 2C). Interaction relationships of the 12 modules were analyzed, and the network heatmap was plotted (Figure 2D). The results revealed that each module was independent from each other, suggesting a high level of independence among the modules and relative independence of genes expression in each module. WGCNA was then used to correlate each module with the four different treatments (including PBS, PHY906, CPT11, and PHY906-CPT11) for the colon tumors GSE25192 dataset by calculating the MS for each module–trait correlation (Figure 2E). After screening for strong correlations between all modules and PHY906, we found the ME in the red module exhibited a higher correlation with PHY906 than other modules (r = 0.58 and *P*=2e-04). Meanwhile, we also found that the pink module had the strongest relationship with CPT11. The black module was positively correlated with the PHY906-CPT11, while the greenyellow module was negatively correlated with the PHY906-CPT11. To identify genes associated with different chemotherapeutic agents of colon cancer, the red module, pink module, greenyellow module, and black module were selected for subsequent research, respectively.

### Functional enrichment analysis, TFs prediction, and identification of hub genes for the red module associated with PHY906

**Figure 3:**
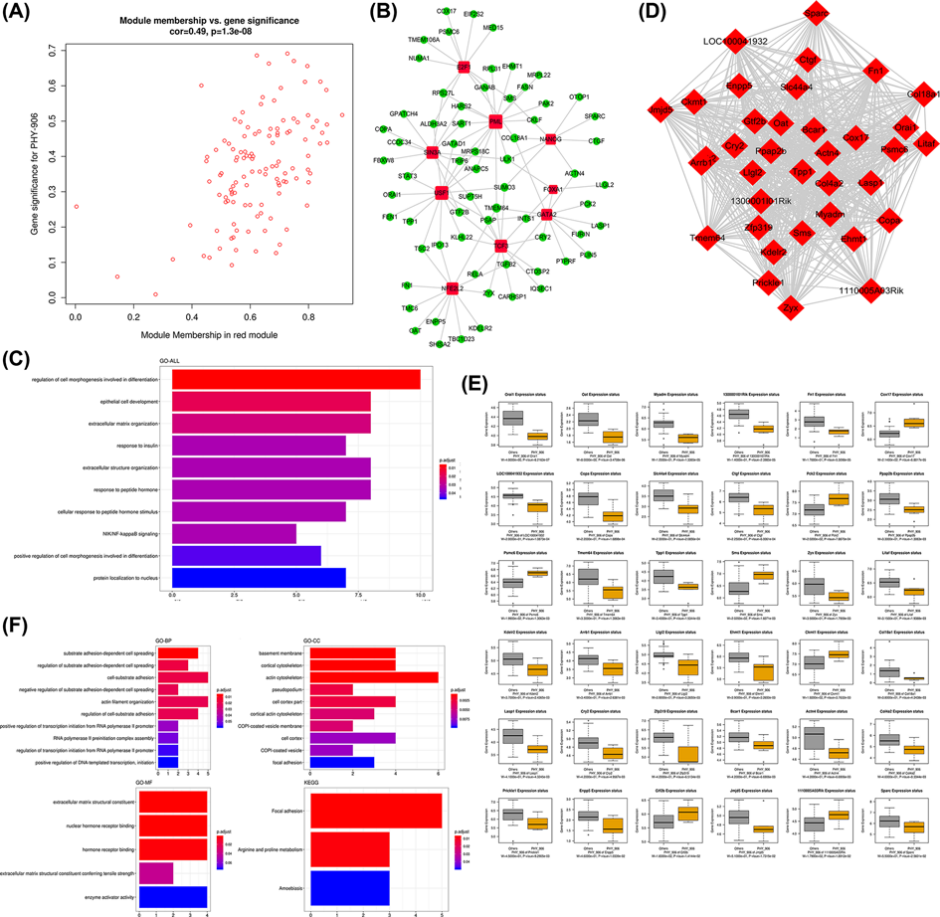
Analysis of the red module negatively associated with PHY906 (**A**) Scatter plot of module eigengenes. (**B**) The TF-target gene regulatory network in the red module (red diamonds represent the TFs, and green nodes represent the genes). (**C**) Functional enrichment analysis for the genes in the red module. (**D**) Network of the hub genes. (**E**) Boxplots of the hub genes between PHY906 and other treatments. (**F**) Functional enrichment analysis for the hub genes.

Scatter plots of GS vs. module membership for the red module was shown in the Figure 3A. Then a total of nine TFs were identified in the red module, which included E2F1, PML, SIN3A, NANOG, USF1, FOXA1, GATA2, TCF3, and NFE2L2. The TF-target gene regulatory network was displayed in Figure 3B. The results of the significant functions and pathways in the red module were presented in Figure 3C. It was found that the genes in this module were associated with regulation of cell morphogenesis involved in differentiation, epithelial cell development, extracellular matrix organization, and so forth. There was no enrichment of this module in KEGG pathway analysis. Afterward, the most significant clusters in the red module was shown in Figure 3D. The results showed that there were 36 hub genes in the module. The boxplots demonstrating the correlations between the tumor treatment and the hub genes were shown in Figure 3E. Except for Cox17, Pck2, Psmc6, Sms, Ckmt1, Gtf2b and 1110005a03rik, the expression status of hub genes in the red module were negatively correlated with PHY906 treatment. According to Figure 3F, the results of GO enrichment analysis revealed the hub genes in red module were significantly enriched in the functional items of substrate adhesion-dependent cell spreading, regulation of substrate adhesion-dependent cell spreading, and cell–substrate adhesion in the category of GO-BP. The results showed the hub genes were significantly enriched in the functional items of basement membrane, cortical cytoskeleton, and actin cytoskeleton in the category of GO-CC. In the category of GO-MF, extracellular matrix structural constituent, nuclear hormone receptor binding, and hormone receptor binding were the representative enrichment terms. For KEGG pathway enrichment analysis, the hub genes in the red module were mainly related to focal adhesion, arginine and proline metabolism, and amoebiasis.

### Functional enrichment analysis, TFs prediction, and identification of hub genes for the pink module associated with CPT11

**Figure 4:**
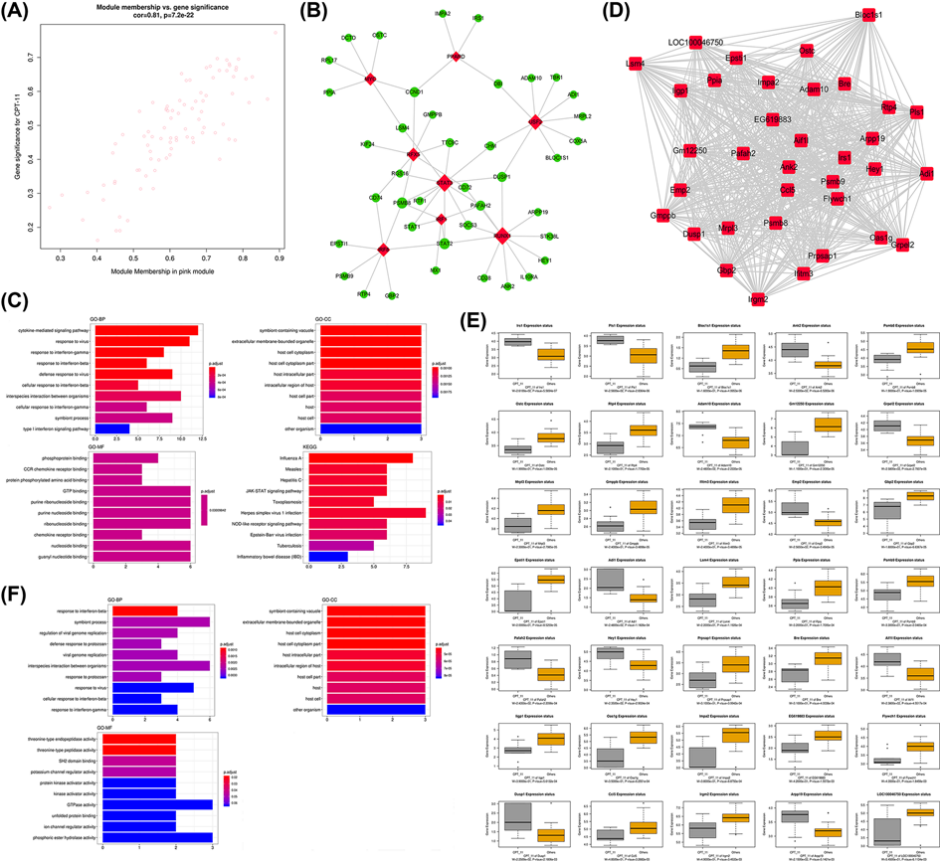
Analysis of the pink module negatively associated with CPT11 (**A**) Scatter plot of module eigengenes. (**B**) The TF-target gene regulatory network in the pink module (red diamonds represent the TFs, and green nodes represent the genes). (**C**) Functional enrichment analysis for the genes in the pink module. (**D**) Network of the hub genes. (**E**) Boxplots of the hub genes between CPT11 and other treatments. (**F**) Functional enrichment analysis for the hub genes.

For the pink module, scatter plots of GS vs. module membership was shown in the Figure 4A. The TF-target gene regulatory network was displayed in Figure 4B, and we identified eight TFs in the module, including MYC, PRARD, RFX5, USF2, STAT3, IRF1, RUNX1, and IRF8. Next, the results of the functional enrichment analysis were listed in Figure 4C. The results showed that the genes in the pink module were significantly enriched in the terms of cytokine-mediated signaling pathway, response to virus, and response IFN-γ in the category of GO-BP, while the category of GO-CC showed that the genes in the pink module were significantly enriched in symbiont-containing vacuole, extracellular membrane-bounded organelle, and host cell cytoplasm. Moreover, phosphoprotein binding, CCR chemokine binding, and protein phosphorylated amino acid binding, and GTP binding were the representative enrichment terms in the category of GO-MF. In KEGG pathway analysis, the genes were significantly involved in several pathway, including influenza A, measles, hepatitis, and JAK-STAT signaling pathway, and so forth. For the pink module, we identified 35 hub genes (Figure 4D). As shown in Figure 4E, all the hub genes were significant in distinguishing CPT11 and other treatments for colon cancers (*P*<0.05), among which Lrs1, Pls1, Ank2, Adam10, Grpel2, Emp2, Adil, Pafah2, Hey1 and Aif1l were up-regulated while Bloclsl, Psmb8, Ostc, Rtp4, Gm12250, Mrpl3, Gm12250, Mrpl3, Gmppb, Lfitm3, Gbp2 and Epstl1 were down-regulated. The results of the GO analysis of the hub genes were shown in Figure 4F, the results revealed the hub genes were significantly enriched in the functional items of response to IFN-β, symbiont process, and regulation of viral genome replication in the category of GO-BP. Then, in the category of GO-CC, the hub genes had significant enrichment in function of symbiont-containing vacuole, extracellular membrane-bounded organelle, and host cell cytoplasm. Moreover, the hub genes were mainly involved in the functional terms of threonine-type endopeptidase activity in the category of GO-MF.

### Functional enrichment analysis, TFs prediction, and identification of hub genes for the black module associated with PHY906-CPT11

**Figure 5:**
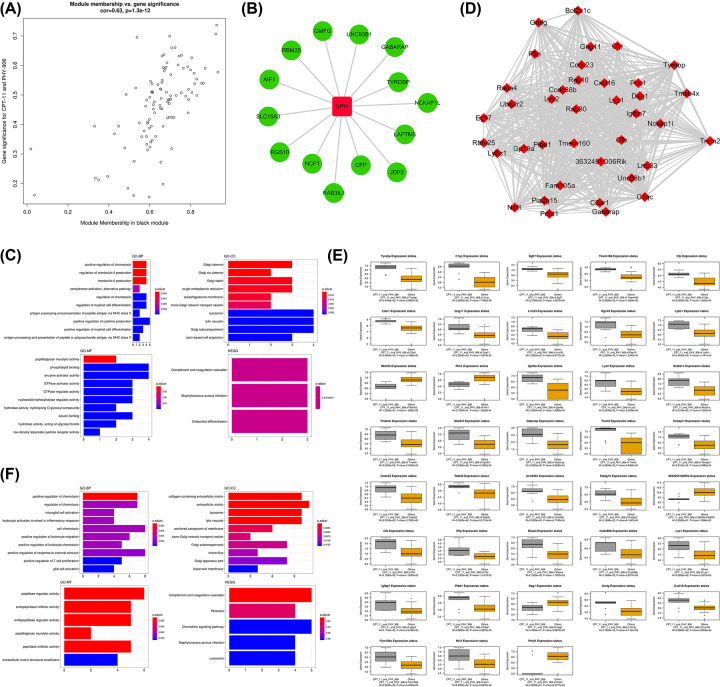
Analysis of the black module positively associated with PHY906-CPT11 (**A**) Scatter plot of module eigengenes. (**B**) The TF-target gene regulatory network in the black module (red diamonds represent the TFs, and green nodes represent the genes). (**C**) Functional enrichment analysis for the genes in the black module. (**D**) Network of the hub genes. (**E**) Boxplots of the hub genes between PHY906-CPT11 and other treatments. (**F**) Functional enrichment analysis for the hub genes.

For the black module, scatter plots of GS vs. module membership was shown in the Figure 5A. There was only one TF identified in the black module. It was SPI1 with 14 target genes. The TF-target gene regulatory network was displayed in Figure 5B. Then, the results of the functional enrichment analysis were listed in Figure 5C. We found genes in the black module most significantly enriched in the functional terms of positive regulation of chemotaxis in the category of GO-BP. In the category of GO-CC, collagen-containing extracellular matrix and extracellular matrix were the representative enrichment terms while peptidase regulator activity, endopeptidase inhibitor activity, endopeptidase regulator activity, and peptidase regulator activity were the most enriched terms in the category of GO-MF. The KEGG analysis revealed the genes in the black module were significantly enriched in the functional items of complement and coagulation cascades. In total 38 hub genes were identified in the black module (Figure 5D). The boxplots demonstrating the correlations between the tumor treatment and the hub genes was shown in Figure 5E. The expression status of Rbm25, Phc1, 3632451o06rik, Dag1, and Prkd were negatively correlated with PHY906-CPT11 treatment, while others had positive correlation. The results of functional enrichment analysis of hub genes in the black module were depicted in Figure 5F. We found the hub genes mainly enriched in positive regulation of chemotaxis, regulation of interleukin-6 production, and interleukin-6 production in the category of GO-BP, and significantly enriched the functional items of Golgi cisterna, Golgi cis cisterna, and Golgi stack in the category of GO-CC. In the category of GO-MF, the genes were mainly involved in peptidoglycan muralytic. In addition, KEGG pathway analysis revealed the hub genes were mainly enriched in the following pathways: complement and coagulation cascades, *Staphylococcus aureus* infection, and osteoclast differentiation.

### Functional enrichment analysis, TFs prediction, and identification of hub genes for the greenyellow module associated with PHY906-CPT11

**Figure 6:**
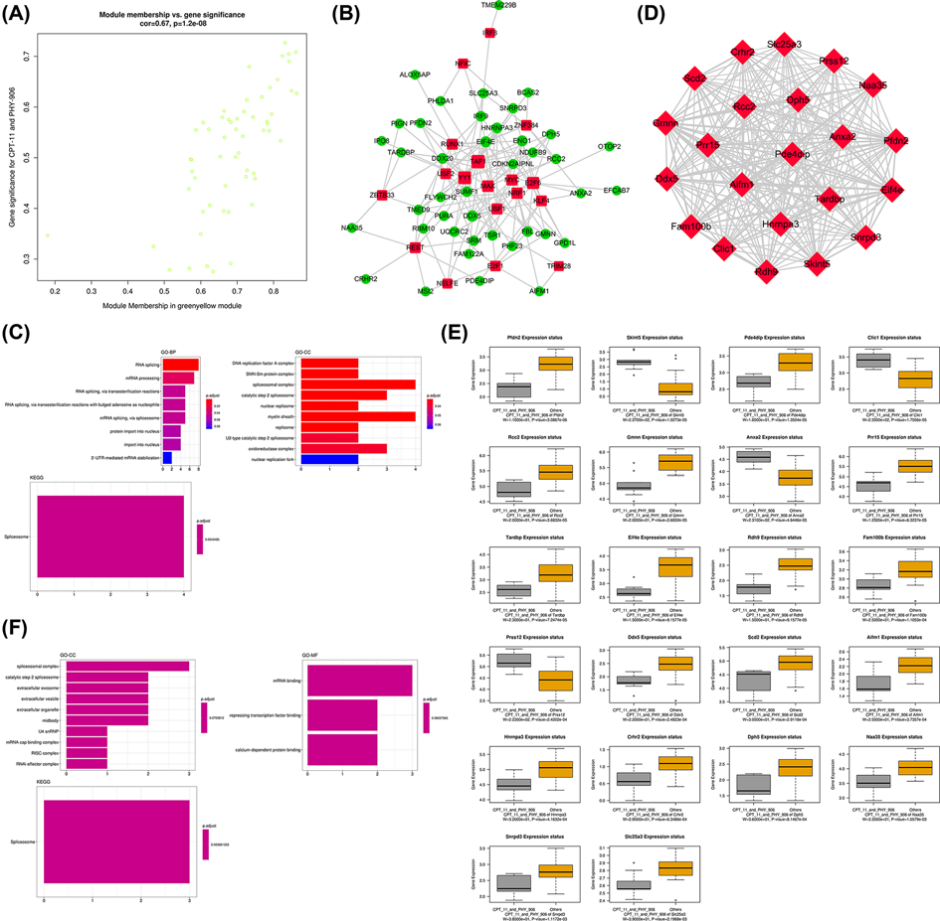
Analysis of the greenyellow module positively associated with PHY906-CPT11 (**A**) Scatter plot of module eigengenes. (**B**) The TF-target gene regulatory network in the greenyellow module (red diamonds represent the TFs, and green nodes represent the genes). (**C**) Functional enrichment analysis for the genes in the greenyellow module. (**D**) Network of the hub genes. (**E**) Boxplots of the hub genes between PHY906-CPT11 and other treatments. (**F**) Functional enrichment analysis for the hub genes.

For the greenyellow module, Scatter plots of GS vs. module membership was shown in the Figure 6A. A total of 18 TFs were identified in the greenyellow module which included IRF8, NFIC, ZBTB33, RUNX1, USF2, REST, TAF1, YY1, ZNF384, MAX, MYC, USF1, NELFE, E2F6, NRF1, KLF4, E2F1 and TRIM28. The TF-target gene regulatory network was displayed in Figure 6B. The results of the functional enrichment analysis were listed in Figure 6C. In the category of GO-BP, we found that the genes in black module were significantly enriched in the functional items of RNA splicing, while the genes in black module were mainly enriched in DNA replication factor A complex, SMN–Sm protein complex, and spliceosomal complex in the category of GO-CC. In the results of KEGG analysis, we found the genes in the black module were significantly enriched in spliceosome. Next, we identified 22 hub genes in the greenyellow module (Figure 6D). As shown in Figure 6E, all the hub genes were significant in distinguishing PHY906-CPT11 from other treatments for colon cancers (*P*<0.05). Except for Skint5, Clic1, Anxa2, and Prss12, the expression status of hub genes in the greenyellow module were negatively correlated with PHY906-CPT11 treatment. The results of functional annotation in the category of GO-CC showed that the hub genes in the greenyellow module were mainly enriched in spliceosomal complex, catalytic step 2 spliceosome, extracellular exosome, and extracellular vesicle. Then, we found that mRNA binding, repressing TF binding, and calcium-dependent protein binding were the representative enrichment in the category of GO-BP. Furthermore, for KEGG analysis, the hub genes in the greenyellow module were significantly enriched in terms of spliceosome (Figure 6F).

### Merging of DEGs with hub genes and their functional enrichment analysis

**Figure 7:**
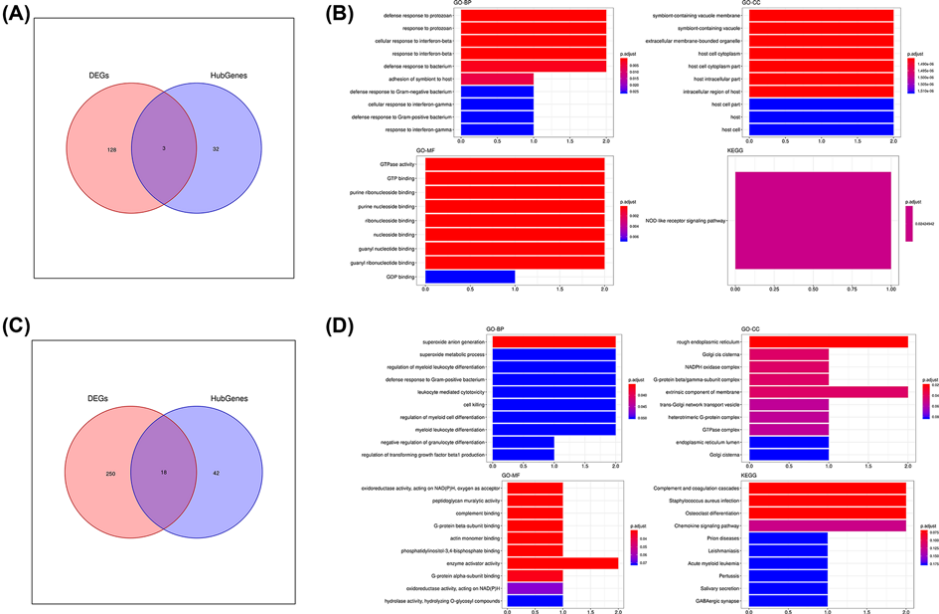
Identification and functional enrichment analysis of key genes (**A**) Venn plot of common genes (key genes) associated with CPT11 treatment. (**B**) GO and KEGG pathway analysis of key genes associated with CPT11 treatment. (**C**) Venn plot of common genes (key genes) associated with PHY906-CPT11 treatment. (**D**) GO and KEGG pathway analysis of key genes associated with PHY906-CPT11 treatment.

In order to further reveal the key genes involved in the treatment of colon cancer with different drugs (PHY906, CPT11, and PHY906-CPT11), we merged the acquired DEGs and the hub genes obtained through WGCNA in different groups, respectively. For PHY906 group, we merged the DEGs with the hub genes associated with PHY906. Then, we found no key genes after the merging. Next, we merged the DEGs acquired from PBS and CPT11 with the hub genes which for the pink module associated with CPT11 (Figure 7A). In total, three key genes were identified which included Ligp1, Gm12250, and Gbp2. As shown in Figure 7B, we performed functional enrichment analysis for the three genes. The enrichment in several GO-BP terms such as defense response to protozoan, response to protozoan, cellular response to IFN-β, response to IFN-β, and defense response to bacterium were detected. In the category of GO-CC, symbiont-containing vacuole membrane, symbiont-containing vacuole, extracellular membrane-bounded organelle, and host cell cytoplasm were the significantly enriched GO term. In the category of GO-MF, GTPase activity, GTP binding, purine bibonucleoside binding and purine nucleoside binding were the most enriched items. As for KEGG pathway analysis, these genes were mostly associated with NOD-like receptor signaling pathway.

For the PHY906-CPT11 group, after merging the DEGs and the hub genes, we finally identified 18 key genes including Eif4e, Prr15, Anxa2, Ddx5, Tardbp, Skint5, Prss12, Hnrnpa3, Pfdn2, Bcl2a1c, Ncf1, Rgs10, C3ar1, Gng11, Tyrobp, Tmsb4x, C1qc, and Lyz2 (Figure 7C). The results of functional enrichment analysis of these key genes are shown in Figure 7D. In the category of GO-BP, superoxide anion generation was the most significantly enriched GO term. The key genes were significantly enriched in the functional items of rough endoplasmic reticulum, Golgi cis cisterna, NADPH oxidase complex, and G-protein β/γ-subunit complex in the category of GO-CC. In the category of GO-MF, we found the key genes had significant enrichment in function of peptidoglycan muralytic activity, complement binding, G-protein β-subunit binding, and actin monomer binding. Furthermore, the results of KEGG analysis revealed the key genes were significantly enriched in terms of complement and coagulation cascades, *Staphylococcus aureus* infection, and osteoclast differentiation.

### Validation of the key genes

Although we finally obtained 21 key genes, 5 of them were the genes of murine. Therefore, we only performed the validation for 16 genes which included GPB2, EIF4E, PRR15, ANXA2, DDX5, TARDBP, PRSS12, HNRNPA3, PFDN2, NCF1, RGS10, C3AR1, GNG11, TYROBP, TMSB4X, and C1QC. For GPB2, a key gene associated with CPT11 treatment, its expression status was significantly different between normal and tumor samples whether in COAD or READ (Supplementary Figure S2A,D). However, expression status of GPB2 was not related to both the tumor stages and overall survival in colon cancer which included COAD and READ (Supplementary Figure S2B,C,E,F). As for the key genes associated with PHY906-CPT11 treatment, we found that EIF4E, PRR15, ANXA2, PRSS12, HNRNPA3, PFDN2, RGS10, GNG11, and TMSB4X showed obviously different expression levels between normal and tumor samples in colon cancer (Supplementary Figures S3 and S4). Besides, as shown in Supplementary Figures S5 and S6, the expression of EIF4E, TYROBP, and PFDN2 were significantly related to the tumor stages in COAD. Next, the results for overall survival analysis was shown in Supplementary Figures S7 and S8, suggesting these 15 key genes might not correlate with OS of colon cancer patients.

**Figure 8:**
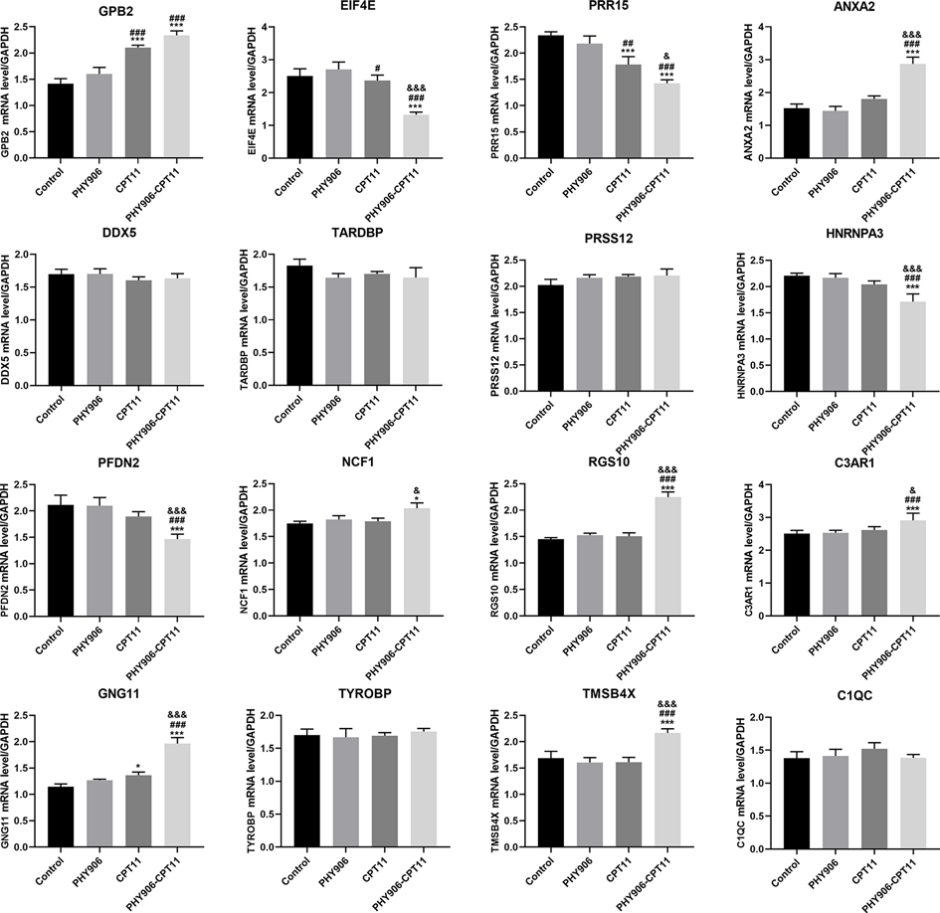
RT-PCR analysis of 16 key genes *P<0.05, ***P<0.005, vs. Control; ^#^*P*<0.05, ^##^*P*<0.01, ^###^*P*<0.005, vs. PHY906, ^&^*P*<0.05, ^&&&^*P*<0.005, vs. CPT11.

To verify the results of the bioinformatics analyses, we conducted qRT-PCR to detect expression of the key genes in SW480 cells after treating with different treatments. As shown in Figure 8, the expression of EIF4E, HNRNPA3, and PFDN2 in SW480 cells of PHY906-CPT11 group were significantly lower than those of other groups (control, PHY906, and CPT11 groups). In the meanwhile, compared with other treatments (PBS or PHY906 or CPT11) the expression of ANXA2, RGS10, GNG11, C3AR1, and TMSB4X in SW480 cells were significantly increased after treating with PHY906-CPT11. Besides, we also found that the expression of GBP2 in CPT11 and PHY906-CPT11 groups were both significantly higher than that in control and PHY906 groups. Collectively, the results of qRT-PCR were similar with the validation results described as above. The EIF4E, HNRNPA3, PFDN2, ANXA2, RGS10, GNG11, C3AR1, and TMSB4X genes might play a role in improving the therapeutic index of CPT11.

## Discussion

Colon cancer is one of the most common causes of cancer deaths worldwide, and its incidence has become higher and higher in recent years. CPT11 is one of the most effective chemotherapeutic drugs for colon cancer patients, but its severe side effects remain unresolved. PHY906 has been commonly used as an adjuvant agent for the treatment of various cancers, especially for colon cancer [29]. Several researches revealed that PHY906 not only enhances the anti-tumor efficacy of CTP11 but also alleviates chemotherapy-induced side effects (diarrhea) in the treatment of advanced colon cancer [30].

In order to provide novel insight to understand the molecular mechanism of different drugs in treating colon cancer, the microarray data from the colon tumors treated with PHY906, CPT11, and PHY906-CPT11 were further analyzed. Initially, we found that only three DEGs were screened out between PBS and PHY906; this might be because PHY906, as an adjuvant in cancer treatment, has no significant effect when treating colon cancer alone [31]. Moreover, 131 DEGs were identified between PBS and CPT11, and 268 DEGs were identified between PBS and PHY906-CPT11, which indicated that the CPT11 and the PHY906-CPT11 administration both induced significant alterations in gene expression levels of colon tumors when compared with PBS treatment. Then, we applied WGCNA to identify the key modules and hub genes in therapy of colon cancer by R and identified four key modules that were most significantly associated with γ three different treatments. Since TFs play important regulatory roles in human diseases, detailed analyses of these regulators will allow us to better understand the molecular mechanisms underlying colon cancer therapy; so, we also constructed TF-gene regulation networks within the key module. The results showed us that the red module mostly correlated with PHY906 treatment in colon cancer, and nine TFs (E2F1, PML, SIN3A, NANOG, USF1, FOXA1, GATA2, TCF3, and NFE2L2) were significantly associated with the genes within red module. Several studies have shown that E2F1 has potential for mediating multiple cancer hallmarks, such as sustaining proliferative signaling, metabolic reprogramming, and tissue invasion and metastasis [32,33]. NANOG is involved in the self-renewal of embryonic stem cells; a previous study indicated that NANOG gene is abnormally overexpressed during the development of malignant phenotype of tumor cells [34]. FOXA1 plays a regulatory role in the evolution and development of organisms, and the recent studies revealed that FOXA1 is associated with a variety of cancers, such as prostate, breast, and gastric [35–37]. After filtering for GS and MM value, we eventually obtained 36 hub genes (Loc100041932, 1300001i01rik, Kdelr2, Lasp1, 1110005a03rik, Llgl2, Copa, Orai1, Bcar1, Actn4, Col4a2, Jmjd5, Arrb1, Cry2, Gtf2b, Tmem64, Zfp319, Myadm, Sparc, Ehmt1, Ctgf, Litaf, Psmc6, Sms, Ckmt1, Oat, Zyx, Col18a1, Cox17, Enpp5, Prickle1, Pck2, Slc44a4, Ppap2b, Fn1, and Tpp1) and their expression were significant in distinguishing PHY906-CPT11 and other treatments for colon cancers. Many studies have reported that some of these 36 genes such as Lasp1, Llgl2, Bcar1, Actn4, Jmjd5, Sparc, Ctgf, Litaf, Ckmt1 and Pck2, are cancer-related genes which function in tumorigenesis and prognosis. As for other hub genes, there are few reports implicating them in cancers. LASP1 is highly expressed in various malignant tumors such as colon cancer and prostate cancer, and is closely related to tumorigenesis, invasion, and metastasis [38,39]. BCAR1 is an inflammatory gene that has been shown to be a novel biomarker for colorectal cancer prognosis [10]. A large number of clinical studies suggested that the changes of ACTN4 expression are related to the aggressiveness, invasiveness, and metastasis in some tumors [40,41]. It has been reported that JMJD5 was important for sustaining cell migration and invasion, and could be a potential oncogene for colon carcinogenesis [42]. SPARC is highly expressed in many tumors, and a research showed that its expression is related with metastasis and recurrence of colon cancer [43,44]. CTGF has shown to be associated with progression, tumorigenesis, and prognosis of various cancers [45,46]. To explore biological functions for the hub genes, we performed the functional enrichment analysis, and the results showed these hub genes were mainly enriched in substrate adhesion-dependent cell spreading and focal adhesion. Focal adhesion is demonstrated to be an essential pathway for BPs including wound healing and tumor metastasis [47]. Furthermore, we merged the DEGs and hub genes which associated with PHY906 treatment and found no common gene. This may be because PHY906 does not have a particularly obvious therapeutic effect on colon cancer, which is similar to the previous DEGs screening results.

For the CPT11 treatment, we identified the pink module as the most significantly related module. Different from the red module associated with PHY906 treatment, in addition to the enrichment in response IFN-γ, the genes in the pink modules were also significantly enriched in cytokine-mediated signaling pathway, and response to virus. In pathway analysis, genes in the pink module was found to be significantly related to JAK-STAT signaling pathway and herpes simplex virus 1 (HSV-1) infection. JAK-STAT pathway is widely involved in cell proliferation, differentiation, apoptosis, and inflammation. The activation of JAK-STAT pathway promotes the occurrence and development of various diseases, including various inflammatory diseases, lymphoma, leukemia, and solid tumors [48,49]. HSV-1 has reportedly been shown to be the most effective oncolytic virus, which can stimulate the body to produce a strong anti-tumor immune response. A previous study demonstrated that HSV-1 has a good clinical effect on colon cancer [50]. TF-gene regulation networks within the pink module had been conducted, in total eight TFs were identified which including MYC, PRARD, RFX5, USF2, STAT3, IRF1, RUNX1, and IRF8. Except for the PRARD, other TFs were previously reported to be related to cancer progression. An increasing body of evidence supports that the cytoplasmic TF STAT3 is activated constitutively in a variety of cancers wherein it significantly affects the growth of tumors and facilitates metastasis [51,52]. USF2 is observed in various human cancers, plays important roles in embryogenesis, metabolism, and cancer development [53]. A previous study indicated that USF2 is associated with tumor grade and inversely with survival in Stage II colon cancers [54]. IRF1 and IRF8 are member of the IFN regulatory factor family; both of them were proven to be suppressor of colon cancer [55,56]. Fijneman and colleagues found a set of RUNX1 target genes in mouse colon indicative of changes in gut homeostasis, that is, genes involved in inflammation and intestinal metabolism, which are known to increase the risk of tumor development [57]. Then, we obtained 35 hub genes within the pink module, and there were 23 cancer-related genes (Lsm4, Arpp19, Rtp4, Ifitm3, Flywch1, Ppia, Bre, Psmb8, Ank2, Mrpl3, Hey1, Impa2, Emp2, Irs1, Psmb9, Epsti1, Ccl5, Bloc1s1, Gbp2, Adam10, Adi1, Aif1l, and Dusp1), which functioned in tumorigenesis and prognosis. FLYWCH1 has the potential to become a biomarker and therapeutic target against metastatic colon cancer [58]. Some studies demonstrated that PPIA expression was up-regulated in all 17 types of cancer assessed when compared with normal tissues [59]. The expression level of IRS1 was found to be higher in colon cancer cells in comparison with the normal cell [60]. Compared with the hub genes associated with PHY906 treatment, there were more cancer-related genes in the hub genes associated with CPT11 treatment. This result may explain why the anti-cancer effect of CPT11 was significantly stronger than that of PHY906. However, the results of the functional enrichment analysis of the 35 hub genes in the pink module seemed to have no obvious correlation with cancer progression. In addition, the overlap of the DEGs with these hub genes allowed the identification of a common list of three key genes which included Ligp1, Gm12250 and Gbp2. Similarly to our results, a previous study demonstrated that GBP2 might be involved in the progression of colon cancer [61]. GO and KEGG pathway enrichment analysis showed that the key genes related to CPT11 treatment were significantly enriched in defense response to protozoan, cellular response to interferon-beta and NOD-like receptor signaling pathway. NOD-like receptor is an inflammatory immune receptor which is connected with many organelles in the cell. In recent years, it is generally believed that inflammation is closely related to tumors, especially chronic inflammation, and even the main driving factor of many tumors [62,63]. Therefore, the treatment of colon cancer with CPT11 is potentially regulated by LIGP1, GM12250 and GBP2, and exerts anti-cancer effects through mediating the NOD-like signaling pathway.

In order to clarify the anti-cancer mechanism of PHY906-CPT11 treatment more clearly, we constructed the WGCNA and screened out the two modules with the most positive and negative correlations. Black module was most positively correlated with PHY906-CPT11 treatment. The genes in the black module were significantly enriched in positive regulation of chemotaxis, which could relate to the cancer invasion and metastasis. Then, a total of 38 hub genes were obtained from the black module. We found that 30 of these genes which included Nckap1l, Ncf1, Pla2g15, Unc93b1, Lrrc33, Rgs10, Gmfg, Rbm25, C3ar1, Ccdc88b, Fam105a, Gng11, Phc1, Rasa4, Dag1, Tyrobp, Tmsb4x, Cfp, Prkd1, Gp49a, Trem2, C1qc, Lyz2, Igfbp7, Rab30, Cxcl16, Egfl7, Ube2r2, Pltp, and Cfb, showed a relation with cancer. Wang and colleagues first assessed GMFG expression in colorectal cancer cell lines and tissue specimens and found the targeting of GMFG expression may suppress colorectal cancer progression [64]. It has been reported that CCDC88B has a critical function in colon inflammation and the pathogenesis of IBD [65], and a previous study proved that patients with IBD have a higher probability of developing colorectal cancer than other people [66]. Strong expression of the CXCL16 protein in primary colon tumor tissue was reported to be correlated to a good prognosis [67]. EGFL7 plays a vital role in controlling vascular angiogenesis during embryogenesis, organogenesis, and maintaining skeletal homeostasis, its dysregulation has been frequently found in several types of cancers [68–70]. Accumulating evidence suggested that EGFL7 plays a crucial role in cancer biology by modulating tumor angiogenesis, metastasis, and invasion [71]. Afterward, we identified the yellowgreen module as the key module which most negatively correlated with PHY906-CPT11 treatment. A total of 18 TFs (IRF8, NFIC, ZBTB33, RUNX1, USF2, REST, TAF1, YY1, ZNF384, MAX, MYC, USF1, NELFE, E2F6, NRF1, KLF4, E2F1, and TRIM28) were identified in this module. Interestingly, we found that E2F1 was also identified in the red module associated with PHY906, while IRF8, RUNX1, and USF2 were also identified in the pink module associated with CPT11. Our study found that genes in the yellowgreen module were significantly enriched in terms of spliceosome. Recently, increasing studies have found that spliceosome has a very important position in the occurrence and treatment of cancer [72]. In the yellowgreen module, a total of 22 hub genes were identified, which included Eif4e, Prr15, Clic1, Anxa2, Ddx5, Tardbp, Pde4dip, Crhr2, Scd2, Skint5, Naa35, Slc25a3, Prss12, Snrpd3, Gmnn, Aifm1, Hnrnpa3, Rcc2, Pfdn2, Rdh9, Dph5, and Fam100b. We found that 17 hub genes were cancer-related genes, which were involved in tumorigenesis and prognosis. Wang and colleagues [73] first demonstrated that CLIC1 regulates the migration and invasion of colon cancer. Several studies found Anxa2 overexpression in different types of tumors including colon cancer [74]. A new research found that ANXA2 mRNA is up-regulated at all stages of colon cancer and ANXA2 protein levels associate with high probability of invasion and distant metastasis [75]. In a previous study, CRHR2 was identified as a gene which contributing to reversal of colorectal cancer cell resistance [76]. Consistent with the result of functional enrichment analysis for the yellowgreen module, the hub genes were also significantly enriched in terms of spliceosome. Compared with the previous two treatment, we found that there were significantly more cancer-related genes involved in PHY906-CPT11 treatment. This is consistent with the results of the comparison of the current treatment effects of the three different treatments, the best in PHY906-CPT11, the second in CPT11, and the worst in PHY906. Finally, we merged the DEGs and the hub genes obtained through WGCNA to find key genes associated with PHY906-CPT11 treatment. A total of 18 key genes were finally obtained, which included EIF4E, PRR15, ANXA2, DDX5, TARDBP, SKINT5, PRSS12, HNRNPA3, PFDN2, BCL2A1C, NCF1, RGS10, C3AR1, GNG11, TYROBP, TMSB4X, C1QC, and LYZ2. Except for SKINT5, other key genes are cancer-related genes and most of these cancer related genes are associated with progression, tumorigenesis, and prognosis of colon cancer. We performed a functional enrichment analysis of key genes, and the results showed that these genes were mainly enriched in the functional terms related to superoxide anion generation, complement and coagulation cascades, and *Staphylococcus aureus* infection. Some studies found an imbalance between the generation of reactive oxygen species (ROS) and the ability of a cancer cell to repair the resulting oxidative damage leads to a state of oxidative stress that can culminate in apoptosis [77]. Complement can mediate immune response and inflammatory response. Complement coagulation cascades is involved in many physiological and pathological processes, including inflammatory processes. Increasing evidence showed that variation in the type and number of intestinal flora plays an important role in the occurrence and development of colorectal cancer [78,79]. Intestinal flora imbalance might induce bacterial infections and inflammation, which can lead to cancer. Therefore, we speculated that these key genes may be the important therapeutic targets for inducing colon cell apoptosis by producing ROS, and inhibiting bacteria and inflammation.

Furthermore, we verified the expression of key genes in TCGA datasets using GEPIA. Before validation of the key genes, we excluded five murine genes which included IIGP1, GM12250, SKINT5, BCL2A1C and LYZ2. The dysregulation of GBP2, EIF4E, PRR15, ANXA2, PRSS12, HNRNPA3, PFDN2, RGS10, GNG11, and TMSB4X were found in the samples of colon cancer, suggesting that these genes are related to progression of colon cancer. In the analysis for relation of gene expression and tumor stages, we found only three of the key genes were significantly related to tumor stages of COAD. Unfortunately, the expression of all key genes we identified were not related to overall survival of colon cancer patients. Besides, we analyzed the expression of 16 key genes by qPCR in SW480 cells treated with different drugs (PHY906, CPT11 and PHY906-CPT11). The results showed that the GBP2, EIF4E, PRR15, ANXA2, HNRNPA3, NCF1, C3AR1, PFDN2, RGS10, GNG11, and TMSB4X were significantly dysregulated in SW480 cells after treating with PHY906-CPT11, which was similar with the above results. Thus, we speculated that PHY906-CPT11 exerts therapeutic role in colon cancer by regulating the expression of GBP2, EIF4E, PRR15, ANXA2, HNRNPA3, NCF1, C3AR1, PFDN2, RGS10, GNG11, and TMSB4X. Previous evidence has indicated that these genes play regulatory roles with other proteins in several tumors including colon cancer [75,80,81].

However, there are some limitations in the present study. First, small sample size limits the statistical power to identify the key genes, but we validated the key genes based on GEPIA and PCR analysis to improve the credibility of our results. Besides, further studies about the genes obtained in the present study are required for elucidating the underlying mechanism of PHY906-CPT11 in colon cancer treatment. Since the NF-κB plays an important role in colon cancer progression, its involvement in PHY906-CPT11 treatment in colon cancer should also be elucidated by *in vivo* studies in future works.

## Conclusions

Nowadays, researchers have increasingly revealed that combination of chemotherapy and TCM would improve the survival of patients, but most of the molecular mechanisms are not clear. In the present study, we applied bioinformatics method to screen out the genes involved in therapeutic process of colon cancer. We identified a total of 18 key genes (EIF4E, PRR15, ANXA2, DDX5, TARDBP, SKINT5, PRSS12, HNRNPA3, PFDN2, BCL2A1C, NCF1, RGS10, C3AR1, GNG11, TYROBP, TMSB4X, C1QC, and LYZ2) associated with PHY906-CPT11 therapy, and found that the expression of EIF4E, PRR15, ANXA2, HNRNPA3, NCF1, C3AR1, PFDN2, RGS10, GNG11, and TMSB4X in SW480 cells was affected by PHY906-CPT11 treatment. Functional enrichment suggested that these key genes might induce cell apoptosis by producing ROS, and inhibit bacteria and inflammation to play a therapeutic role in colon cancer. Although our investigation is of a preliminary nature and further research is needed to verify these findings, our work might provide direction for specific molecular mechanisms of combination of chemotherapy and TCM in future studies.

## Supplementary Material

Supplementary Figures S1-S8

## Data Availability

All data generated or analyzed during the present study are included in this published article.

## Abbreviations

BMI: body mass index
BP: biological process
CCR: C-C chemokine receptor
COAD: colon adenocarcinoma
CPT11: Irinotecan
DEG: differentially expressed gene
FC: fold changes
FDR: false discovery rate
GAPDH: Glyceraldehyde-3-phosphate dehydrogenase
GEO: Gene Expression Omnibus
GEPIA: Gene Expression Profiling Interactive Analysis
GO: gene ontology
GS: gene significance
HSV-1: herpes simplex virus 1
IBD: inflammatory bowel disease
IFN: interferon
KEGG: Kyoto Encyclopedia of Genes and Genomes
MCODE: Molecular Complex Detection
ME: module eigengene
MM: module membership
NOD: Nucleotide binding oligomerization domain
PBS: phosphate buffered saline
qRT-PCR: quantitative reverse transcription polymerase chain reaction
READ: rectal adenocarcinoma
ROS: reactive oxygen species
RT-PCR: reverse transcription polymerase chain reaction
TCGA: The Cancer Genome Atlas
TCM: traditional Chinese medicine
TF: transcription factor
WGCNA: weighted gene co-expression network analysis
Z.K: standardized connectivity

## References

[1] Bray F., Ferlay J., Soerjomataram I., Siegel R.L., Torre L.A. and Jemal A. (2018) Global cancer statistics 2018: GLOBOCAN estimates of incidence and mortality worldwide for 36 cancers in 185 countries. CA Cancer J. Clin. 68, 394–424 10.3322/caac.21492 30207593

[2] Johnson C.M., Wei C., Ensor J.E., Smolenski D.J., Amos C.I., Levin B. et al. (2013) Meta-analyses of colorectal cancer risk factors. Cancer Causes Control 24, 1207–1222 10.1007/s10552-013-0201-5 23563998 PMC4161278

[3] Tsoi H., Chu E.S.H., Zhang X., Sheng J., Nakatsu G., Ng S.C. et al. (2017) Peptostreptococcus anaerobius induces intracellular cholesterol biosynthesis in colon cells to induce proliferation and causes dysplasia in mice. Gastroenterology 152, 1419.e5–1433.e5 10.1053/j.gastro.2017.01.009 28126350

[4] Wang X., Wang J., Rao B. and Deng L. (2017) Gut flora profiling and fecal metabolite composition of colorectal cancer patients and healthy individuals. Exp. Ther. Med. 13, 2848–2854 10.3892/etm.2017.4367 28587349 PMC5450625

[5] Tahara T., Yamamoto E., Suzuki H., Maruyama R., Chung W., Garriga J. et al. (2014) Fusobacterium in colonic flora and molecular features of colorectal carcinoma. Cancer Res. 74, 1311–1318 10.1158/0008-5472.CAN-13-1865 24385213 PMC4396185

[6] Ito M., Kanno S., Nosho K., Sukawa Y., Mitsuhashi K., Kurihara H. et al. (2015) Association of Fusobacterium nucleatum with clinical and molecular features in colorectal serrated pathway. Int. J. Cancer 137, 1258–1268 10.1002/ijc.29488 25703934

[7] Qu D., Shen L., Liu S., Li H., Ma Y., Zhang R. et al. (2017) Chronic inflammation confers to the metabolic reprogramming associated with tumorigenesis of colorectal cancer. Cancer Biol. Ther. 18, 237–244 10.1080/15384047.2017.1294292 28278072 PMC5450783

[8] Waldner M.J. and Neurath M.F. (2014) Master regulator of intestinal disease: IL-6 in chronic inflammation and cancer development. Semin. Immunol. 26, 75–79 10.1016/j.smim.2013.12.003 24447345

[9] Li J., Wang H., Zheng Z., Luo L., Wang P., Liu K. et al. (2018) Mkp-1 cross-talks with Nrf2/Ho-1 pathway protecting against intestinal inflammation. Free Radic. Biol. Med. 124, 541–549 10.1016/j.freeradbiomed.2018.07.002 30061089

[10] Jiang H., Dong L., Gong F., Gu Y., Zhang H., Fan D. et al. (2018) Inflammatory genes are novel prognostic biomarkers for colorectal cancer. Int. J. Mol. Med. 42, 368–380 29693170 10.3892/ijmm.2018.3631 PMC5979867

[11] Fujita K., Kubota Y., Ishida H. and Sasaki Y. (2015) Irinotecan, a key chemotherapeutic drug for metastatic colorectal cancer. World J. Gastroenterol. 21, 12234–12248 10.3748/wjg.v21.i43.12234 26604633 PMC4649109

[12] Shiozawa T., Tadokoro J., Fujiki T., Fujino K., Kakihata K., Masatani S. et al. (2013) Risk factors for severe adverse effects and treatment-related deaths in Japanese patients treated with irinotecan-based chemotherapy: a postmarketing survey. Jpn. J. Clin. Oncol. 43, 483–491 10.1093/jjco/hyt040 23536639 PMC3638635

[13] Gao J., Zhou J., Li Y., Peng Z., Li Y., Wang X. et al. (2013) Associations between UGT1A1*6/*28 polymorphisms and irinotecan-induced severe toxicity in Chinese gastric or esophageal cancer patients. Med. Oncol. 30, 630 10.1007/s12032-013-0630-8 23783485

[14] Bazarbashi S., Aljubran A., Alzahrani A., Mohieldin A., Soudy H. and Shoukri M. (2015) Phase I/II trial of capecitabine, oxaliplatin, and irinotecan in combination with bevacizumab in first line treatment of metastatic colorectal cancer. Cancer Med. 4, 1505–1513 10.1002/cam4.497 26207614 PMC4618621

[15] Zou Y., Lin J., Li W., Wu Z., He Z., Huang G. et al. (2016) Huangqin-tang ameliorates dextran sodium sulphate-induced colitis by regulating intestinal epithelial cell homeostasis, inflammation and immune response. Sci. Rep. 6, 39299 10.1038/srep39299 27982094 PMC5159883

[16] Huang S., Peng W., Mao D., Zhang S., Xu P., Yi P. et al. (2019) Kangai injection, a traditional Chinese medicine, improves efficacy and reduces toxicity of chemotherapy in advanced colorectal cancer patients: a systematic review and meta-analysis. Evid. Based Complement. Alternat. Med. 2019, 8423037 10.1155/2019/8423037 31379968 PMC6662435

[17] Ye L., Jia Y., Ji K.E., Sanders A.J., Xue K., Ji J. et al. (2015) Traditional Chinese medicine in the prevention and treatment of cancer and cancer metastasis. Oncol. Lett. 10, 1240–1250 10.3892/ol.2015.3459 26622657 PMC4533180

[18] Wu J., Liu Y., Fang C., Zhao L., Lin L. and Lu L. (2019) Traditional Chinese medicine preparation combined therapy may improve chemotherapy efficacy: a systematic review and meta-analysis. Evid. Based Complement. Alternat. Med. 2019, 5015824 10.1155/2019/5015824 31320914 PMC6610742

[19] Wang E., Bussom S., Chen J., Quinn C., Bedognetti D., Lam W. et al. (2011) Interaction of a traditional Chinese Medicine (PHY906) and CPT-11 on the inflammatory process in the tumor microenvironment. BMC Med. Genet. 4, 38 10.1186/1755-8794-4-38 21569348 PMC3117677

[20] Liang J.W., Fang Z.Y., Huang Y., Liuyang Z.Y., Zhang X.L., Wang J.L. et al. (2018) Application of Weighted Gene Co-Expression Network Analysis to explore the key genes in Alzheimer’s disease. J. Alzheimers Dis. 65, 1353–1364 10.3233/JAD-180400 30124448 PMC6218130

[21] Feltrin A.S., Tahira A.C., Simões S.N., Brentani H. and Martins D.C. Jr (2019) Assessment of complementarity of WGCNA and NERI results for identification of modules associated to schizophrenia spectrum disorders. PLoS ONE 14, e0210431 10.1371/journal.pone.0210431 30645614 PMC6333352

[22] Yang Q., Wang R., Wei B., Peng C., Wang L., Hu G. et al. (2018) Candidate biomarkers and molecular mechanism investigation for glioblastoma multiforme utilizing WGCNA. Biomed. Res. Int. 2018, 4246703 10.1155/2018/4246703 30356407 PMC6178162

[23] Liu R., Zhang W., Liu Z.Q. and Zhou H.H. (2017) Associating transcriptional modules with colon cancer survival through weighted gene co-expression network analysis. BMC Genomics 18, 361 10.1186/s12864-017-3761-z 28486948 PMC5424422

[24] Xue K., Cao J., Wang Y., Zhao X., Yu D., Jin C. et al. (2020) Identification of potential therapeutic gene markers in nasopharyngeal carcinoma based on bioinformatics analysis. Clin. Transl. Sci. 13, 265–274 10.1111/cts.12690 31863646 PMC7070980

[25] Xiong Y., Yuan L., Xiong J., Xu H., Luo Y., Wang G. et al. (2020) An outcome model for human bladder cancer: a comprehensive study based on weighted gene co-expression network analysis. J. Cell. Mol. Med. 24, 2342–2355 10.1111/jcmm.14918 31883309 PMC7011142

[26] Ritchie M.E., Phipson B., Wu D., Hu Y., Law C.W., Shi W. et al. (2015) limma powers differential expression analyses for RNA-sequencing and microarray studies. Nucleic Acids Res. 43, e47 10.1093/nar/gkv007 25605792 PMC4402510

[27] Langfelder P. and Horvath S. (2008) WGCNA: an R package for weighted correlation network analysis. BMC Bioinformatics 9, 559 10.1186/1471-2105-9-559 19114008 PMC2631488

[28] Livak K.J. and Schmittgen T.D. (2001) Analysis of relative gene expression data using real-time quantitative PCR and the 2(-Delta Delta C(T)) Method. Methods 25, 402–408 10.1006/meth.2001.1262 11846609

[29] Liu S.H. and Cheng Y.C. (2012) Old formula, new Rx: the journey of PHY906 as cancer adjuvant therapy. J. Ethnopharmacol. 140, 614–623 10.1016/j.jep.2012.01.047 22326673

[30] Lam W., Jiang Z., Guan F., Hu R., Liu S.H., Chu E. et al. (2014) The number of intestinal bacteria is not critical for the enhancement of antitumor activity and reduction of intestinal toxicity of irinotecan by the Chinese herbal medicine PHY906 (KD018). BMC Complement. Alternat. Med. 14, 490 10.1186/1472-6882-14-490 25510341 PMC4302098

[31] Lam W., Jiang Z., Guan F., Huang X., Hu R., Wang J. et al. (2015) PHY906(KD018), an adjuvant based on a 1800-year-old Chinese medicine, enhanced the anti-tumor activity of Sorafenib by changing the tumor microenvironment. Sci. Rep. 5, 9384 10.1038/srep09384 25819872 PMC4377583

[32] Zhou Q., Wang C., Zhu Y., Wu Q., Jiang Y., Huang Y. et al. (2019) Key genes and pathways controlled By E2F1 in human castration-resistant prostate cancer cells. Onco Targets Ther. 12, 8961–8976 10.2147/OTT.S217347 31802906 PMC6827506

[33] Malaney P., Palumbo E., Semidey-Hurtado J., Hardee J., Stanford K., Kathiriya J.J. et al. (2018) PTEN physically interacts with and regulates E2F1-mediated transcription in lung cancer. Cell Cycle 17, 947–962 10.1080/15384101.2017.1388970 29108454 PMC6103743

[34] Muinao T., Deka Boruah H.P. and Pal M. (2018) Diagnostic and prognostic biomarkers in ovarian cancer and the potential roles of cancer stem cells - an updated review. Exp. Cell Res. 362, 1–10 10.1016/j.yexcr.2017.10.018 29079264

[35] Lin M., Pan J., Chen Q., Xu Z., Lin X. and Shi C. (2018) Overexpression of FOXA1 inhibits cell proliferation and EMT of human gastric cancer AGS cells. Gene 642, 145–151 10.1016/j.gene.2017.11.023 29129808

[36] Rangel N., Fortunati N., Osella-Abate S., Annaratone L., Isella C., Catalano M.G. et al. (2018) FOXA1 and AR in invasive breast cancer: new findings on their co-expression and impact on prognosis in ER-positive patients. BMC Cancer 18, 703 10.1186/s12885-018-4624-y 29970021 PMC6029370

[37] Song B., Park S.H., Zhao J.C., Fong K.W., Li S., Lee Y. et al. (2019) Targeting FOXA1-mediated repression of TGF-β signaling suppresses castration-resistant prostate cancer progression. J. Clin. Invest. 129, 569–582 10.1172/JCI122367 30511964 PMC6355239

[38] Zhou R., Shao Z., Liu J., Zhan W., Gao Q., Pan Z. et al. (2018) COPS5 and LASP1 synergistically interact to downregulate 14-3-3σ expression and promote colorectal cancer progression via activating PI3K/AKT pathway. Int. J. Cancer 142, 1853–1864 10.1002/ijc.31206 29226323

[39] Nishikawa R., Goto Y., Sakamoto S., Chiyomaru T., Enokida H., Kojima S. et al. (2014) Tumor-suppressive microRNA-218 inhibits cancer cell migration and invasion via targeting of LASP1 in prostate cancer. Cancer Sci. 105, 802–811 10.1111/cas.12441 24815849 PMC4317931

[40] Wang N., Wang Q., Tang H., Zhang F., Zheng Y., Wang S. et al. (2017) Direct inhibition of ACTN4 by ellagic acid limits breast cancer metastasis via regulation of β-catenin stabilization in cancer stem cells. J. Exp. Clin. Cancer Res. 36, 172 10.1186/s13046-017-0635-9 29197410 PMC5712102

[41] Watanabe T., Ueno H., Watabe Y., Hiraoka N., Morizane C., Itami J. et al. (2015) ACTN4 copy number increase as a predictive biomarker for chemoradiotherapy of locally advanced pancreatic cancer. Br. J. Cancer 112, 704–713 10.1038/bjc.2014.623 25602965 PMC4333489

[42] Zhang R., Huang Q., Li Y., Song Y. and Li Y. (2015) JMJD5 is a potential oncogene for colon carcinogenesis. Int. J. Clin. Exp. Pathol. 8, 6482–6489 26261525 PMC4525859

[43] Drev D., Harpain F., Beer A., Stift A., Gruber E.S., Klimpfinger M. et al. (2019) Impact of fibroblast-derived SPARC on invasiveness of colorectal cancer cells. Cancers (Basel) 11, 1421 10.3390/cancers11101421 31554208 PMC6827058

[44] Zhai X., Xue Q., Liu Q., Guo Y. and Chen Z. (2017) Colon cancer recurrence-associated genes revealed by WGCNA co-expression network analysis. Mol. Med. Rep. 16, 6499–6505 10.3892/mmr.2017.7412 28901407 PMC5865817

[45] Wells J.E., Howlett M., Cole C.H. and Kees U.R. (2015) Deregulated expression of connective tissue growth factor (CTGF/CCN2) is linked to poor outcome in human cancer. Int. J. Cancer 137, 504–511 10.1002/ijc.28972 24832082

[46] Lun W., Wu X., Deng Q. and Zhi F. (2018) MiR-218 regulates epithelial-mesenchymal transition and angiogenesis in colorectal cancer via targeting CTGF. Cancer Cell Int. 18, 83 10.1186/s12935-018-0575-2 29977158 PMC5994014

[47] Beri P., Popravko A., Yeoman B., Kumar A., Chen K., Hodzic E. et al. (2020) Cell adhesiveness serves as a biophysical marker for metastatic potential. Cancer Res. 80, 901–911 10.1158/0008-5472.CAN-19-1794 31857292 PMC7024658

[48] Thomas S.J., Snowden J.A., Zeidler M.P. and Danson S.J. (2015) The role of JAK/STAT signalling in the pathogenesis, prognosis and treatment of solid tumours. Br. J. Cancer 113, 365–371 10.1038/bjc.2015.233 26151455 PMC4522639

[49] Villarino A.V., Kanno Y. and O’Shea J.J. (2017) Mechanisms and consequences of Jak-STAT signaling in the immune system. Nat. Immunol. 18, 374–384 10.1038/ni.3691 28323260 PMC11565648

[50] Kolodkin-Gal D., Edden Y., Hartshtark Z., Ilan L., Khalaileh A., Pikarsky A.J. et al. (2009) Herpes simplex virus delivery to orthotopic rectal carcinoma results in an efficient and selective antitumor effect. Gene Ther. 16, 905–915 10.1038/gt.2009.44 19440231

[51] Kryczek I., Lin Y., Nagarsheth N., Peng D., Zhao L., Zhao E. et al. (2014) IL-22(+)CD4(+) T cells promote colorectal cancer stemness via STAT3 transcription factor activation and induction of the methyltransferase DOT1L. Immunity 40, 772–784 10.1016/j.immuni.2014.03.010 24816405 PMC4032366

[52] Chai E.Z., Shanmugam M.K., Arfuso F., Dharmarajan A., Wang C., Kumar A.P. et al. (2016) Targeting transcription factor STAT3 for cancer prevention and therapy. Pharmacol. Ther. 162, 86–97 26478441 10.1016/j.pharmthera.2015.10.004

[53] Chi T.F., Horbach T., Götz C., Kietzmann T. and Dimova E.Y. (2019) Cyclin-dependent kinase 5 (CDK5)-mediated phosphorylation of upstream stimulatory factor 2 (USF2) contributes to carcinogenesis. Cancers (Basel) 11, 10.3390/cancers11040523 PMC6521020 31013770

[54] Christensen L.L., Tobiasen H., Holm A., Schepeler T., Ostenfeld M.S., Thorsen K. et al. (2013) MiRNA-362-3p induces cell cycle arrest through targeting of E2F1, USF2 and PTPN1 and is associated with recurrence of colorectal cancer. Int. J. Cancer 133, 67–78 10.1002/ijc.28010 23280316

[55] AbuSara N., Razavi S., Derwish L., Komatsu Y., Licursi M. and Hirasawa K. (2015) Restoration of IRF1-dependent anticancer effects by MEK inhibition in human cancer cells. Cancer Lett. 357, 575–581 10.1016/j.canlet.2014.12.017 25497010

[56] Ibrahim M.L., Klement J.D., Lu C., Redd P.S., Xiao W., Yang D. et al. (2018) Myeloid-derived suppressor cells produce IL-10 to elicit DNMT3b-dependent IRF8 silencing to promote colitis-associated colon tumorigenesis. Cell Rep. 25, 3036.e6–3046.e6 10.1016/j.celrep.2018.11.050 30540937 PMC6319669

[57] Fijneman R.J., Anderson R.A., Richards E., Liu J., Tijssen M., Meijer G.A. et al. (2012) Runx1 is a tumor suppressor gene in the mouse gastrointestinal tract. Cancer Sci. 103, 593–599 10.1111/j.1349-7006.2011.02189.x 22171576 PMC5439111

[58] Muhammad B.A., Almozyan S., Babaei-Jadidi R., Onyido E.K., Saadeddin A., Kashfi S.H. et al. (2018) FLYWCH1, a novel suppressor of nuclear β-Catenin, regulates migration and morphology in colorectal cancer. Mol. Cancer Res. 16, 1977–1990 10.1158/1541-7786.MCR-18-0262 30097457 PMC6277001

[59] Cheng S., Luo M., Ding C., Peng C., Lv Z., Tong R. et al. (2016) Downregulation of Peptidylprolyl isomerase A promotes cell death and enhances doxorubicin-induced apoptosis in hepatocellular carcinoma. Gene 591, 236–244 10.1016/j.gene.2016.07.020 27397650

[60] Bailey K.L., Agarwal E., Chowdhury S., Luo J., Brattain M.G., Black J.D. et al. (2017) TGFβ/Smad3 regulates proliferation and apoptosis through IRS-1 inhibition in colon cancer cells. PLoS ONE 12, e0176096 10.1371/journal.pone.0176096 28414818 PMC5393866

[61] Liao C., Huang X., Gong Y. and Lin Q. (2019) Discovery of core genes in colorectal cancer by weighted gene co-expression network analysis. Oncol. Lett. 18, 3137–3149 31402962 10.3892/ol.2019.10605 PMC6676736

[62] Kim Y.K., Shin J.S. and Nahm M.H. (2016) NOD-like receptors in infection, immunity, and diseases. Yonsei Med. J. 57, 5–14 10.3349/ymj.2016.57.1.5 26632377 PMC4696971

[63] Velloso F.J., Trombetta-Lima M., Anschau V., Sogayar M.C. and Correa R.G. (2019) NOD-like receptors: major players (and targets) in the interface between innate immunity and cancer. Biosci. Rep. 39, BSR20181709 10.1042/BSR20181709 30837326 PMC6454022

[64] Wang H., Chen Z., Chang H. et al. (2017) Expression of glia maturation factor γ is associated with colorectal cancer metastasis and its downregulation suppresses colorectal cancer cell migration and invasion in vitro. Oncol Rep 37, 929–936 10.3892/or.2017.5361 28075454

[65] Fodil N., Moradin N., Leung V., Olivier J.F., Radovanovic I., Jeyakumar T. et al. (2017) CCDC88B is required for pathogenesis of inflammatory bowel disease. Nat. Commun. 8, 932 10.1038/s41467-017-01381-y 29030607 PMC5640600

[66] Anzai H., Hata K., Kishikawa J., Ishii H., Nishikawa T., Tanaka T. et al. (2016) Clinical pattern and progression of ulcerative proctitis in the Japanese population: a retrospective study of incidence and risk factors influencing progression. Colorectal Dis. 18, O97–O102 10.1111/codi.13237 26663677

[66] AbdelMageed M., Ali H., Olsson L., Lindmark G., Hammarström M.L., Hammarström S. et al. (2019) The chemokine CXCL16 is a new biomarker for lymph node analysis of colon cancer outcome. Int. J. Mol. Sci. 20, 5793 10.3390/ijms20225793 31752131 PMC6888697

[68] Deng Q.J., Xie L.Q. and Li H. (2016) Overexpressed MALAT1 promotes invasion and metastasis of gastric cancer cells via increasing EGFL7 expression. Life Sci. 157, 38–44 10.1016/j.lfs.2016.05.041 27259812

[69] Pannier D., Philippin-Lauridant G., Baranzelli M.C., Bertin D., Bogart E., Delprat V. et al. (2016) High expression levels of egfl7 correlate with low endothelial cell activation in peritumoral vessels of human breast cancer. Oncol. Lett. 12, 1422–1428 10.3892/ol.2016.4791 27446447 PMC4950557

[70] Dudvarski Stanković N., Bicker F., Keller S., Jones D.T., Harter P.N., Kienzle A. et al. (2018) EGFL7 enhances surface expression of integrin α(5)β(1) to promote angiogenesis in malignant brain tumors. EMBO Mol. Med. 10, 10.15252/emmm.201708420 PMC6127886 30065025

[71] Hong G., Kuek V., Shi J., Zhou L., Han X., He W. et al. (2018) EGFL7: Master regulator of cancer pathogenesis, angiogenesis and an emerging mediator of bone homeostasis. J. Cell. Physiol. 233, 8526–8537 10.1002/jcp.26792 29923200

[72] Hsu T.Y., Simon L.M., Neill N.J., Marcotte R., Sayad A., Bland C.S. et al. (2015) The spliceosome is a therapeutic vulnerability in MYC-driven cancer. Nature 525, 384–388 10.1038/nature14985 26331541 PMC4831063

[73] Wang P., Zeng Y., Liu T., Zhang C., Yu P.W., Hao Y.X. et al. (2014) Chloride intracellular channel 1 regulates colon cancer cell migration and invasion through ROS/ERK pathway. World J. Gastroenterol. 20, 2071–2078 10.3748/wjg.v20.i8.2071 24587680 PMC3934477

[74] Liu X., Ma D., Jing X., Wang B., Yang W. and Qiu W. (2015) Overexpression of ANXA2 predicts adverse outcomes of patients with malignant tumors: a systematic review and meta-analysis. Med. Oncol. 32, 392 10.1007/s12032-014-0392-y 25476478

[75] Rocha M.R., Barcellos-de-Souza P., Sousa-Squiavinato A.C.M., Fernandes P.V., de Oliveira I.M., Boroni M. et al. (2018) Annexin A2 overexpression associates with colorectal cancer invasiveness and TGF-ß induced epithelial mesenchymal transition via Src/ANXA2/STAT3. Sci. Rep. 8, 11285 10.1038/s41598-018-29703-0 30050103 PMC6062537

[76] Pothoulakis C., Torre-Rojas M., Duran-Padilla M.A., Gevorkian J., Zoras O., Chrysos E. et al. (2018) CRHR2/Ucn2 signaling is a novel regulator of miR-7/YY1/Fas circuitry contributing to reversal of colorectal cancer cell resistance to Fas-mediated apoptosis. Int. J. Cancer 142, 334–346 10.1002/ijc.31064 28929494 PMC5918308

[77] Moloney J.N. and Cotter T.G. (2018) ROS signalling in the biology of cancer. Semin. Cell Dev. Biol. 80, 50–64 28587975 10.1016/j.semcdb.2017.05.023

[78] Chung M., York B.R. and Michaud D.S. (2019) Oral health and cancer. Curr. Oral Health Rep. 6, 130–137 10.1007/s40496-019-0213-7 31871854 PMC6927401

[79] Wong S.H. and Yu J. (2019) Gut microbiota in colorectal cancer: mechanisms of action and clinical applications. Nat. Rev. Gastroenterol. Hepatol. 16, 690–704 31554963 10.1038/s41575-019-0209-8

[80] Xi C., Wang L., Yu J., Ye H., Cao L. and Gong Z. (2018) Inhibition of eukaryotic translation initiation factor 4E is effective against chemo-resistance in colon and cervical cancer. Biochem. Biophys. Res. Commun. 503, 2286–2292 10.1016/j.bbrc.2018.06.150 29959920

[81] Meunier D., Patra K., Smits R., Hägebarth A., Lüttges A., Jaussi R. et al. (2011) Expression analysis of proline rich 15 (Prr15) in mouse and human gastrointestinal tumors. Mol. Carcinog. 50, 8–15 10.1002/mc.20692 21061267

