# Supplementary material for: WGCNA reveals key gene modules regulated by the combined treatment of colon cancer with PHY906 and CPT11
Source: Biosci Rep. 2020 Sep 2;40(9):BSR20200935. doi: 10.1042/BSR20200935 (PMC7468096; doi:10.1042/BSR20200935)
Supplement: Supplementary Figures S1-S8 [file BSR-2020-0935_supp.pdf]

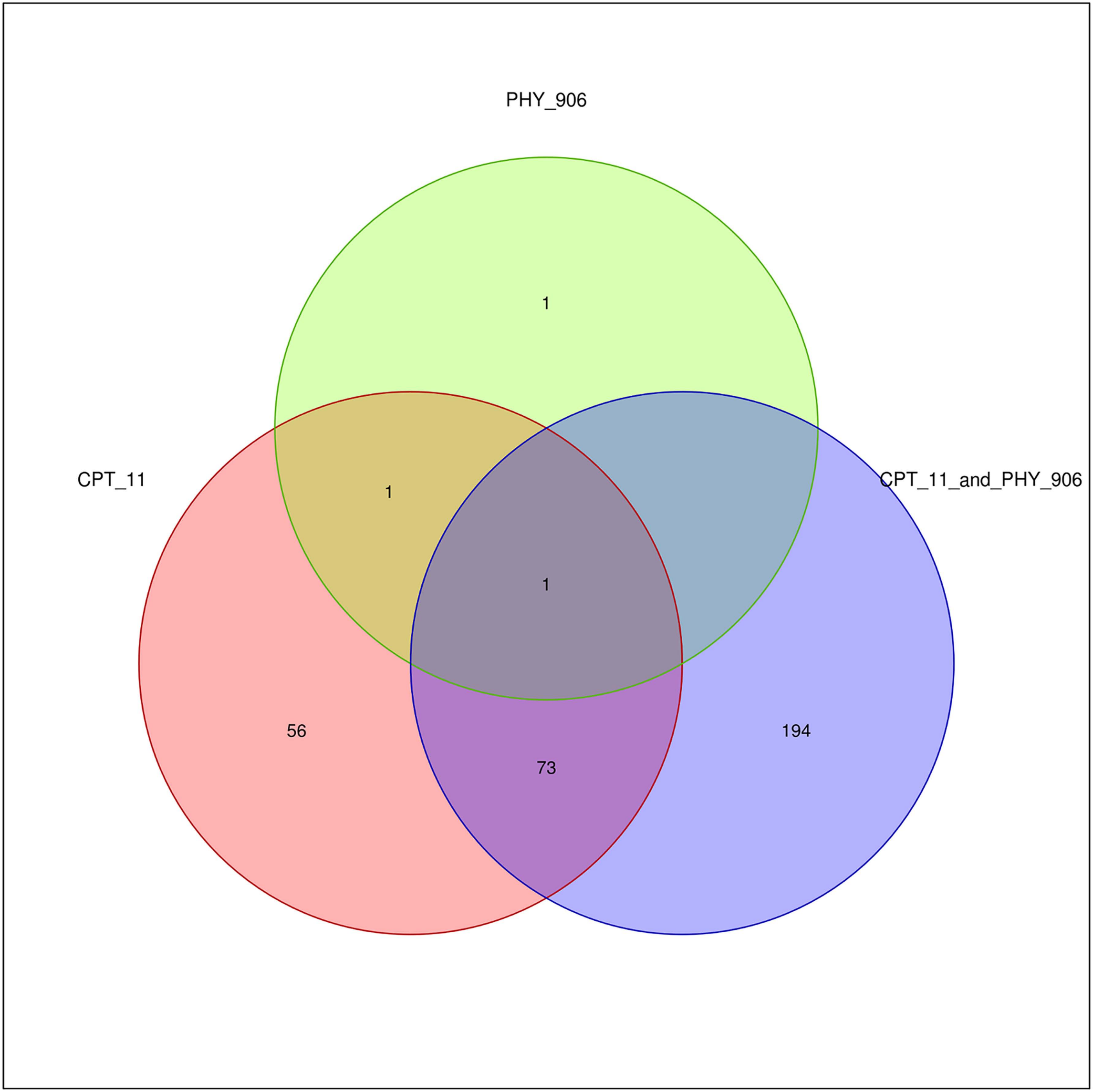
PHY_906
CPT_11
CPT_11_and_PHY_906
1
1
1
56
73
194

Figure S1. Venn plot of common genes between three groups of DEGs.

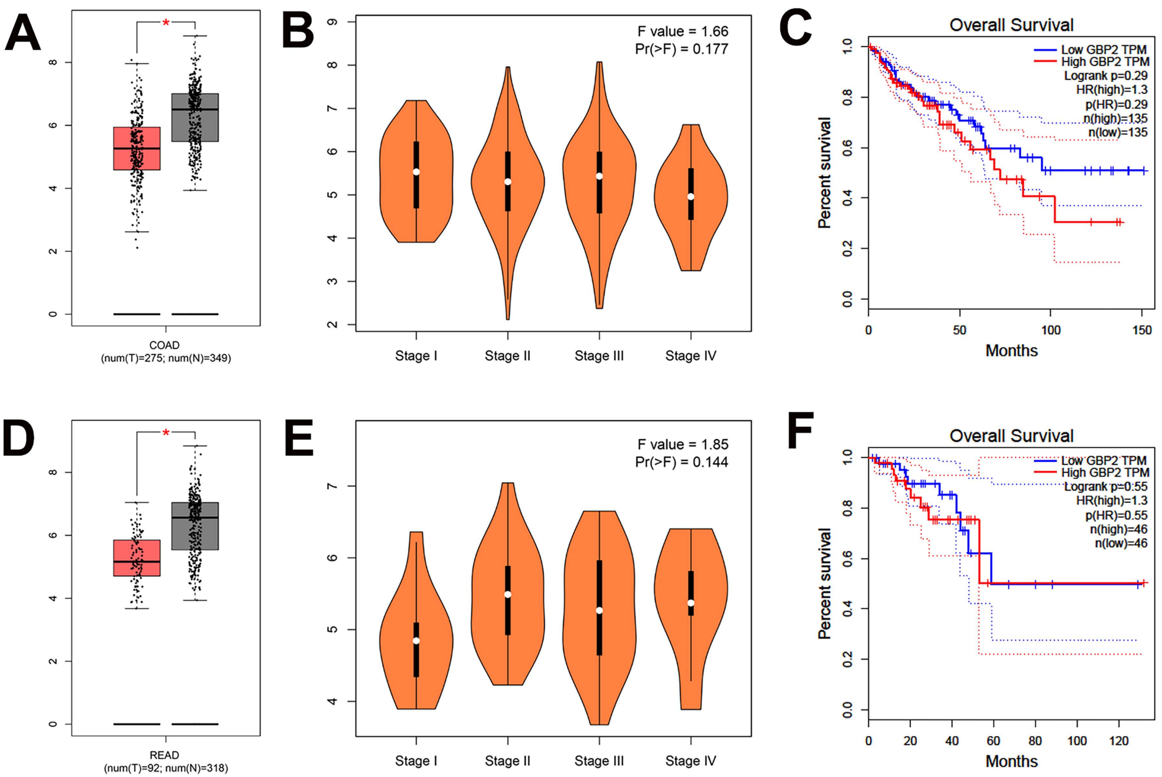
A
COAD
(num(T)=275; num(N)=349)
B
F value = 1.66
Pr(>F) = 0.177
Stage I
Stage II
Stage III
Stage IV
C
Overall Survival
Low GBP2 TPM
High GBP2 TPM
Logrank p=0.29
HR(high)=1.3
p(HR)=0.29
n(high)=135
n(low)=135
Percent survival
Months
D
READ
(num(T)=92; num(N)=318)
E
F value = 1.85
Pr(>F) = 0.144
Stage I
Stage II
Stage III
Stage IV
F
Overall Survival
Low GBP2 TPM
High GBP2 TPM
Logrank p=0.55
HR(high)=1.3
p(HR)=0.55
n(high)=46
n(low)=46
Percent survival
Months

Figure S2. Validation of GBP2.GBP2 expression differences between tumor and normal tissues in **(A)** COAD and **(D)** READ; GBP2 expression in tumor samples with different stages in **(B)** COAD and **(E)** READ; overall survival analysis of GBP2 in **(C)**COAD and **(F)** READ.

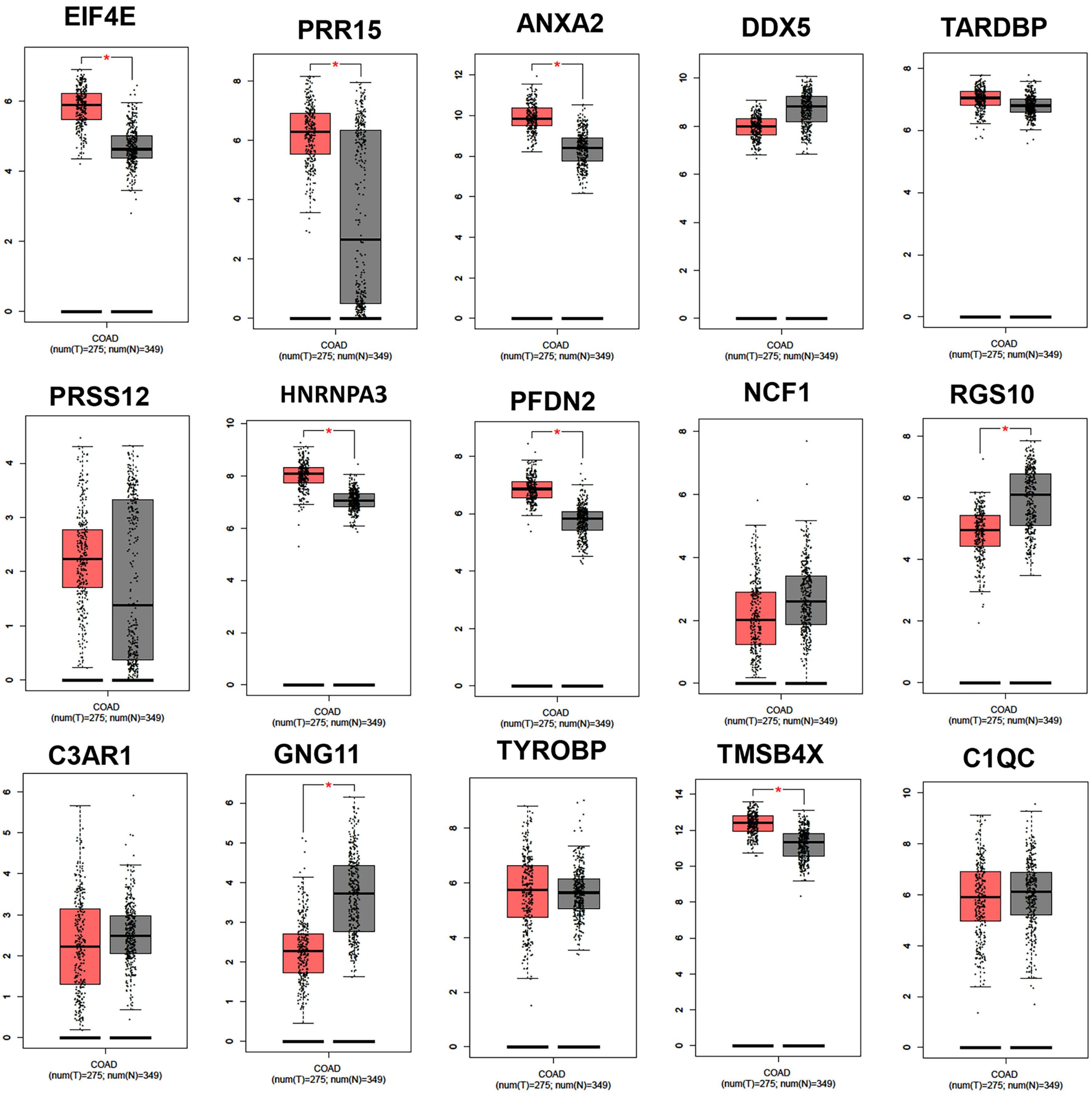

EIF4E
PRR15
ANXA2
DDX5
TARDBP
PRSS12
HNRNPA3
PFDN2
NCF1
RGS10
C3AR1
GNG11
TYROBP
TMSB4X
C1QC
COAD
(num(T)=275; num(N)=349)

Figure S3. Gene expression of 15 key genes associated with PHY906-CPT11 treatment between tumor and normal tissues in COAD. The red box corresponds to tumor samples and the grey box corresponds to normal samples. The vertical axis represents the relative expression of genes. * P <0.05.

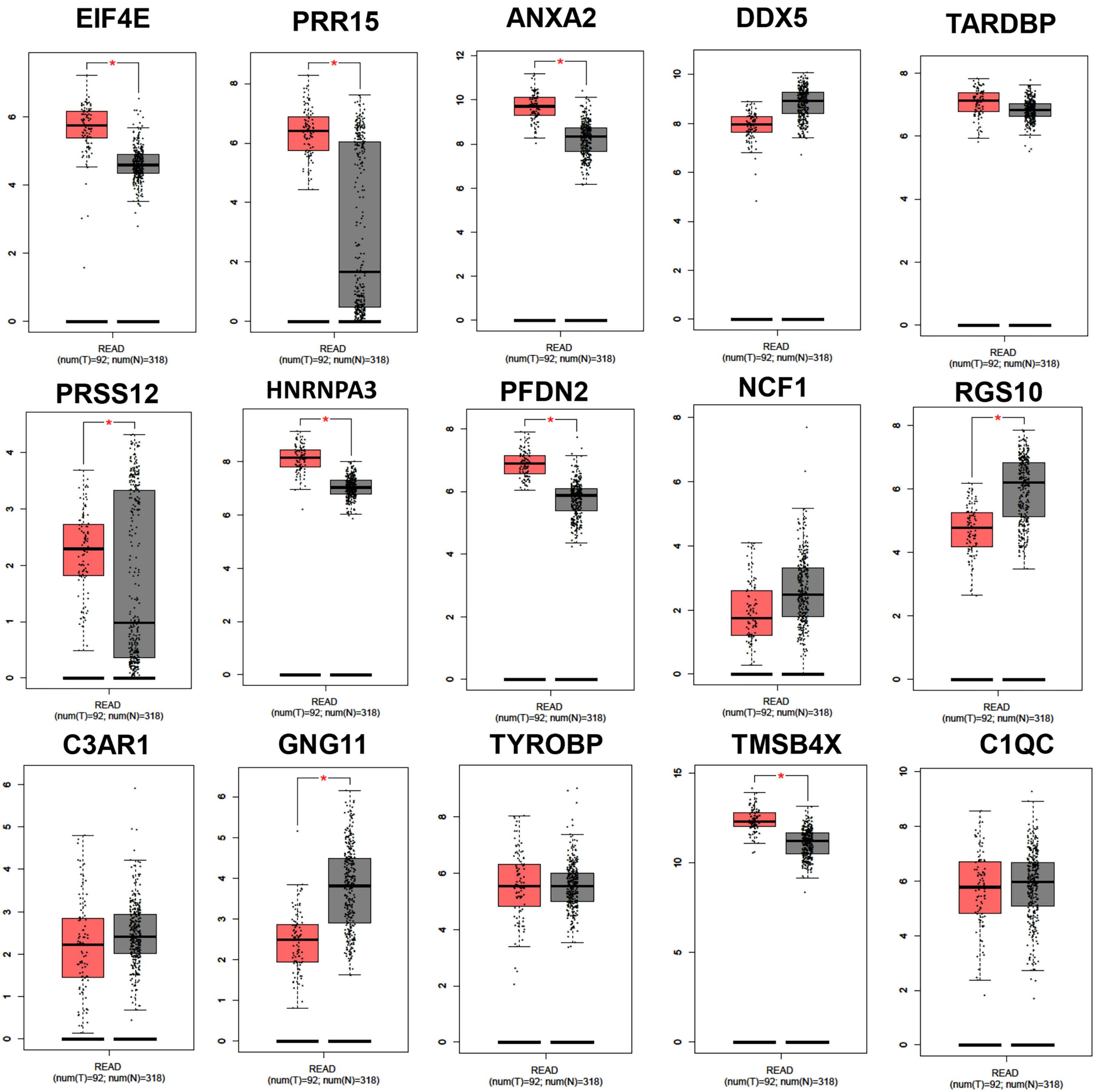
EIF4E
READ
(num(T)=92; num(N)=318)
PRR15
READ
(num(T)=92; num(N)=318)
ANXA2
READ
(num(T)=92; num(N)=318)
DDX5
READ
(num(T)=92; num(N)=318)
TARDBP
READ
(num(T)=92; num(N)=318)
PRSS12
READ
(num(T)=92; num(N)=318)
HNRNPA3
READ
(num(T)=92; num(N)=318)
PFDN2
READ
(num(T)=92; num(N)=318)
NCF1
READ
(num(T)=92; num(N)=318)
RGS10
READ
(num(T)=92; num(N)=318)
C3AR1
READ
(num(T)=92; num(N)=318)
GNG11
READ
(num(T)=92; num(N)=318)
TYROBP
READ
(num(T)=92; num(N)=318)
TMSB4X
READ
(num(T)=92; num(N)=318)
C1QC
READ
(num(T)=92; num(N)=318)

Figure S4. Gene expression of 15 key genes associated with PHY906-CPT11 treatment between tumor and normal tissues in READ. The red box corresponds to tumor samples and the grey box corresponds to non-tumor samples. The vertical axis represents the relative expression of genes. * $P < 0.05$.

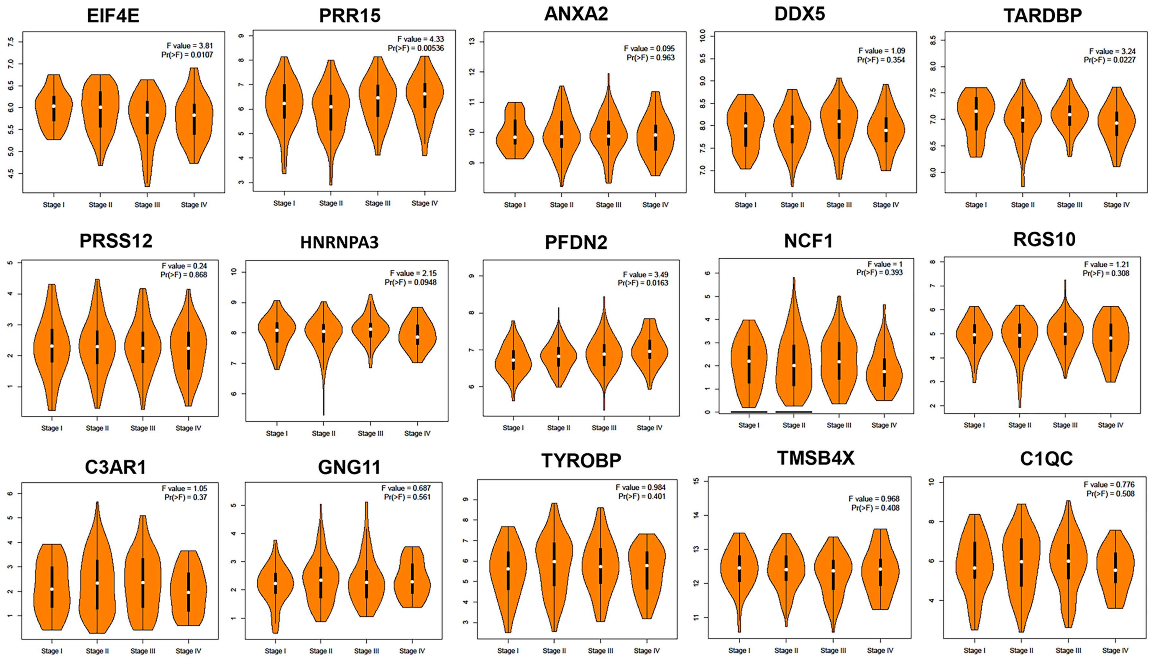
EIF4E
F value = 3.81
Pr(>F) = 0.0107
PRR15
F value = 4.33
Pr(>F) = 0.00536
ANXA2
F value = 0.095
Pr(>F) = 0.963
DDX5
F value = 1.09
Pr(>F) = 0.354
TARDBP
F value = 3.24
Pr(>F) = 0.0227
PRSS12
F value = 0.24
Pr(>F) = 0.868
HNRNPA3
F value = 2.15
Pr(>F) = 0.0948
PFDN2
F value = 3.49
Pr(>F) = 0.0163
NCF1
F value = 1
Pr(>F) = 0.393
RGS10
F value = 1.21
Pr(>F) = 0.308
C3AR1
F value = 1.05
Pr(>F) = 0.37
GNG11
F value = 0.687
Pr(>F) = 0.561
TYROBP
F value = 0.984
Pr(>F) = 0.401
TMSB4X
F value = 0.968
Pr(>F) = 0.408
C1QC
F value = 0.776
Pr(>F) = 0.508
Stage I
Stage II
Stage III
Stage IV

Figure S5. Expression of 15 key genes associated with PHY906-CPT11 treatment in tumor samples with different stages in COAD.

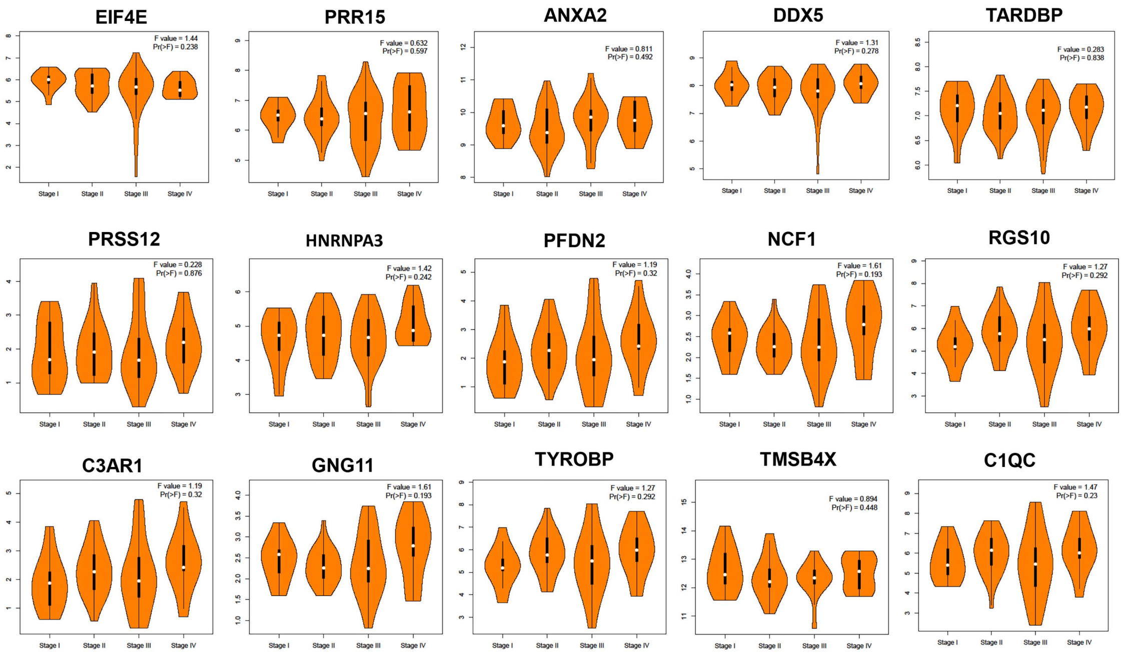
EIF4E
F value = 1.44
Pr(>F) = 0.238
PRR15
F value = 0.632
Pr(>F) = 0.597
ANXA2
F value = 0.811
Pr(>F) = 0.492
DDX5
F value = 1.31
Pr(>F) = 0.278
TARDBP
F value = 0.283
Pr(>F) = 0.838
PRSS12
F value = 0.228
Pr(>F) = 0.876
HNRNPA3
F value = 1.42
Pr(>F) = 0.242
PFDN2
F value = 1.19
Pr(>F) = 0.32
NCF1
F value = 1.61
Pr(>F) = 0.193
RGS10
F value = 1.27
Pr(>F) = 0.292
C3AR1
F value = 1.19
Pr(>F) = 0.32
GNG11
F value = 1.61
Pr(>F) = 0.193
TYROBP
F value = 1.27
Pr(>F) = 0.292
TMSB4X
F value = 0.894
Pr(>F) = 0.448
C1QC
F value = 1.47
Pr(>F) = 0.23
Stage I
Stage II
Stage III
Stage IV

Figure S6. Expression of 15 key genes associated with PHY906-CPT11 treatment in tumor samples with different stages in READ.

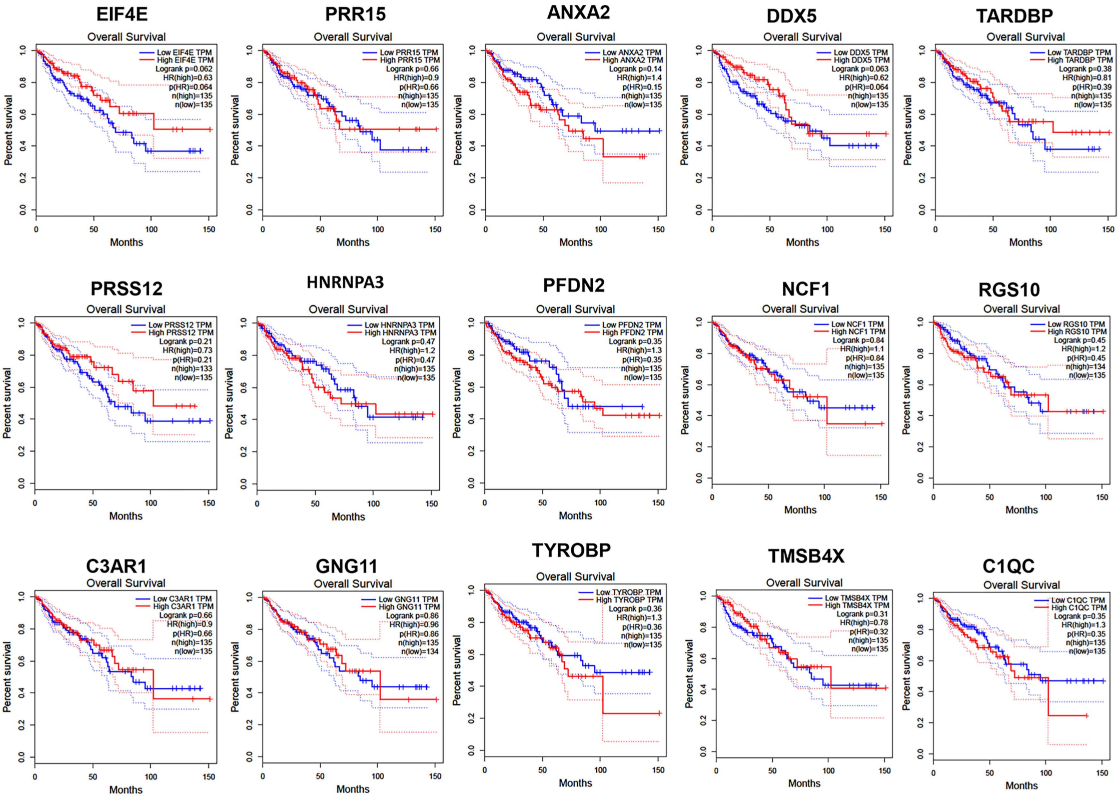
EIF4E
Overall Survival
Low EIF4E TPM
High EIF4E TPM
Logrank p=0.062
HR(high)=0.63
p(HR)=0.064
n(high)=135
n(low)=135
PRR15
Overall Survival
Low PRR15 TPM
High PRR15 TPM
Logrank p=0.66
HR(high)=0.9
p(HR)=0.66
n(high)=135
n(low)=135
ANXA2
Overall Survival
Low ANXA2 TPM
High ANXA2 TPM
Logrank p=0.14
HR(high)=1.4
p(HR)=0.15
n(high)=135
n(low)=135
DDX5
Overall Survival
Low DDX5 TPM
High DDX5 TPM
Logrank p=0.063
HR(high)=0.62
p(HR)=0.064
n(high)=135
n(low)=135
TARDBP
Overall Survival
Low TARDBP TPM
High TARDBP TPM
Logrank p=0.38
HR(high)=0.81
p(HR)=0.39
n(high)=135
n(low)=135
PRSS12
Overall Survival
Low PRSS12 TPM
High PRSS12 TPM
Logrank p=0.21
HR(high)=0.73
p(HR)=0.21
n(high)=133
n(low)=135
HNRNPA3
Overall Survival
Low HNRNPA3 TPM
High HNRNPA3 TPM
Logrank p=0.47
HR(high)=1.2
p(HR)=0.47
n(high)=135
n(low)=135
PFDN2
Overall Survival
Low PFDN2 TPM
High PFDN2 TPM
Logrank p=0.35
HR(high)=1.3
p(HR)=0.35
n(high)=135
n(low)=135
NCF1
Overall Survival
Low NCF1 TPM
High NCF1 TPM
Logrank p=0.84
HR(high)=1.1
p(HR)=0.84
n(high)=135
n(low)=135
RGS10
Overall Survival
Low RGS10 TPM
High RGS10 TPM
Logrank p=0.45
HR(high)=1.2
p(HR)=0.45
n(high)=134
n(low)=135
C3AR1
Overall Survival
Low C3AR1 TPM
High C3AR1 TPM
Logrank p=0.66
HR(high)=0.9
p(HR)=0.66
n(high)=135
n(low)=135
GNG11
Overall Survival
Low GNG11 TPM
High GNG11 TPM
Logrank p=0.86
HR(high)=0.96
p(HR)=0.86
n(high)=135
n(low)=134
TYROBP
Overall Survival
Low TYROBP TPM
High TYROBP TPM
Logrank p=0.36
HR(high)=1.3
p(HR)=0.36
n(high)=135
n(low)=135
TMSB4X
Overall Survival
Low TMSB4X TPM
High TMSB4X TPM
Logrank p=0.31
HR(high)=0.78
p(HR)=0.32
n(high)=135
n(low)=135
C1QC
Overall Survival
Low C1QC TPM
High C1QC TPM
Logrank p=0.35
HR(high)=1.3
p(HR)=0.35
n(high)=135
n(low)=135
Percent survival
Months

Figure S7. Overall survival analyses of 15 key genes associated with PHY906-CPT11 treatment in COAD.

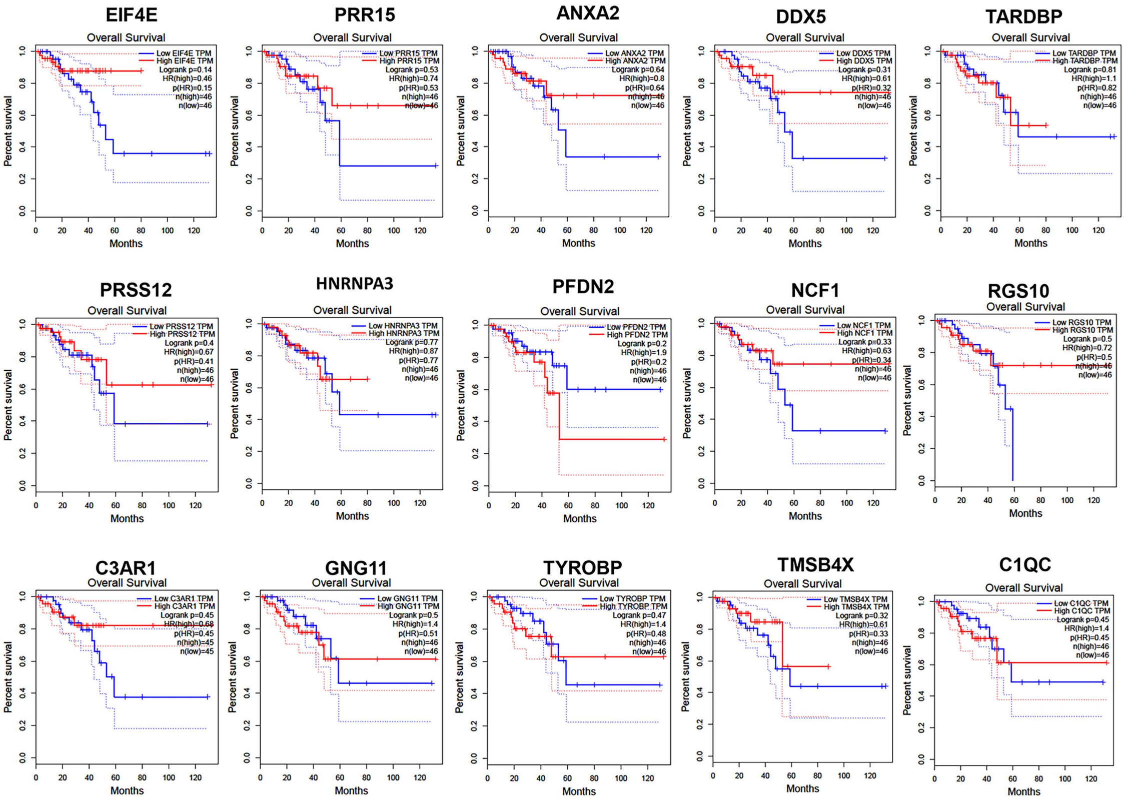

EIF4E
Overall Survival
Low EIF4E TPM
High EIF4E TPM
Logrank p=0.14
HR(high)=0.46
p(HR)=0.15
n(high)=46
n(low)=46
PRR15
Overall Survival
Low PRR15 TPM
High PRR15 TPM
Logrank p=0.53
HR(high)=0.74
p(HR)=0.53
n(high)=46
n(low)=46
ANXA2
Overall Survival
Low ANXA2 TPM
High ANXA2 TPM
Logrank p=0.64
HR(high)=0.8
p(HR)=0.64
n(high)=46
n(low)=46
DDX5
Overall Survival
Low DDX5 TPM
High DDX5 TPM
Logrank p=0.31
HR(high)=0.61
p(HR)=0.32
n(high)=46
n(low)=46
TARDBP
Overall Survival
Low TARDBP TPM
High TARDBP TPM
Logrank p=0.81
HR(high)=1.1
p(HR)=0.82
n(high)=46
n(low)=46
PRSS12
Overall Survival
Low PRSS12 TPM
High PRSS12 TPM
Logrank p=0.4
HR(high)=0.67
p(HR)=0.41
n(high)=46
n(low)=46
HNRNPA3
Overall Survival
Low HNRNPA3 TPM
High HNRNPA3 TPM
Logrank p=0.77
HR(high)=0.87
p(HR)=0.77
n(high)=46
n(low)=46
PFDN2
Overall Survival
Low PFDN2 TPM
High PFDN2 TPM
Logrank p=0.2
HR(high)=1.9
p(HR)=0.2
n(high)=46
n(low)=46
NCF1
Overall Survival
Low NCF1 TPM
High NCF1 TPM
Logrank p=0.33
HR(high)=0.63
p(HR)=0.34
n(high)=46
n(low)=46
RGS10
Overall Survival
Low RGS10 TPM
High RGS10 TPM
Logrank p=0.5
HR(high)=0.72
p(HR)=0.5
n(high)=46
n(low)=46
C3AR1
Overall Survival
Low C3AR1 TPM
High C3AR1 TPM
Logrank p=0.45
HR(high)=0.68
p(HR)=0.45
n(high)=45
n(low)=45
GNG11
Overall Survival
Low GNG11 TPM
High GNG11 TPM
Logrank p=0.5
HR(high)=1.4
p(HR)=0.51
n(high)=46
n(low)=46
TYROBP
Overall Survival
Low TYROBP TPM
High TYROBP TPM
Logrank p=0.47
HR(high)=1.4
p(HR)=0.48
n(high)=46
n(low)=46
TMSB4X
Overall Survival
Low TMSB4X TPM
High TMSB4X TPM
Logrank p=0.32
HR(high)=0.61
p(HR)=0.33
n(high)=46
n(low)=46
C1QC
Overall Survival
Low C1QC TPM
High C1QC TPM
Logrank p=0.45
HR(high)=1.4
p(HR)=0.45
n(high)=46
n(low)=46
Percent survival
Months
0.0
0.2
0.4
0.6
0.8
1.0
0
20
40
60
80
100
120

Figure S8. Overall survival analyses of 15 key genes associated with PHY906-CPT11 treatment in READ.
